# Supplementary material for: Bio-inspired vertebral design for scalable and flexible perovskite solar cells
Source: Nat Commun. 2020 Jun 15;11:3016. doi: 10.1038/s41467-020-16831-3 (PMC7295992; doi:10.1038/s41467-020-16831-3)
Supplement: Supplementary file 1 — Supplementary Information [file 41467_2020_16831_MOESM1_ESM.pdf]

SUPPLEMENTARY INFORMATION

Bio-inspired vertebral design for scalable and flexible perovskite solar cells

Xiangchuan Meng[1,2], Zheren Cai[3], Yanyan Zhang[3], Xiaotian Hu[*1,2], Zhi Xing[1], Zengqi Huang[1], Zhandong Huang[3], Yongjie Cui[5], Ting Hu[1,2], Meng Su[3], Xunfan Liao[5,6], Lin Zhang[7], Fuyi Wang[3], Yanlin Song[*3], Yiwang Chen[*1,2,6]

[1]College of Chemistry, Nanchang University, 999 Xuefu Avenue, Nanchang 330031, China.

[2]Institute of Polymers and Energy Chemistry, Nanchang University, 999 Xuefu Avenue, Nanchang 330031, China.

[3]Key Laboratory of Green Printing, Institute of Chemistry, Chinese Academy of Sciences (ICCAS), Beijing 100190, China.

[4]CAS Key Laboratory of Analytical Chemistry for Living Biosystems, Institute of Chemistry, Chinese Academy of Sciences (ICCAS), Beijing 100190, China.

[5]College of Materials Science and Engineering, Donghua University, Shanghai 201620, China.

[6]Institute of Advanced Scientific Research (iASR), Jiangxi Normal University, 99 Ziyang Avenue, Nanchang 330022, China.

[7]Hunan Key Laboratory of Super Microstructure and Ultrafast Process, School of Physics and Electronics, Central South University, Changsha 410083, China.

[*]E-mail: ywchen@ncu.edu.cn (Y.W.C.); ylsong@iccas.ac.cn (Y.S.); xiaotian@iccas.ac.cn (X.H.)

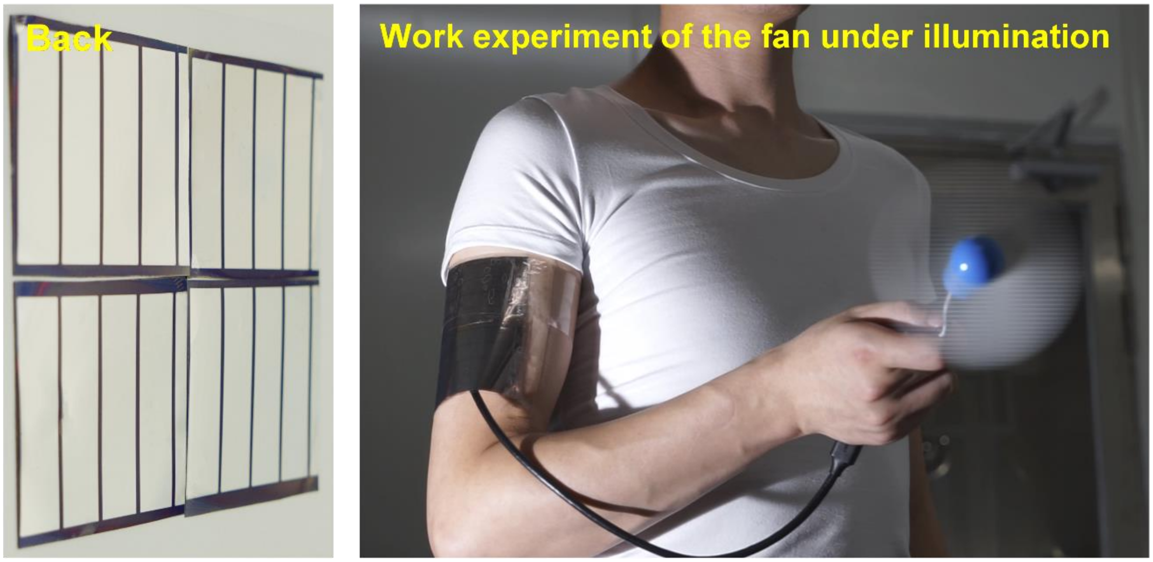

Supplementary Figure 1. The photographs of flexible PSMs prepared by meniscus-coating and the corresponding wearable applications.

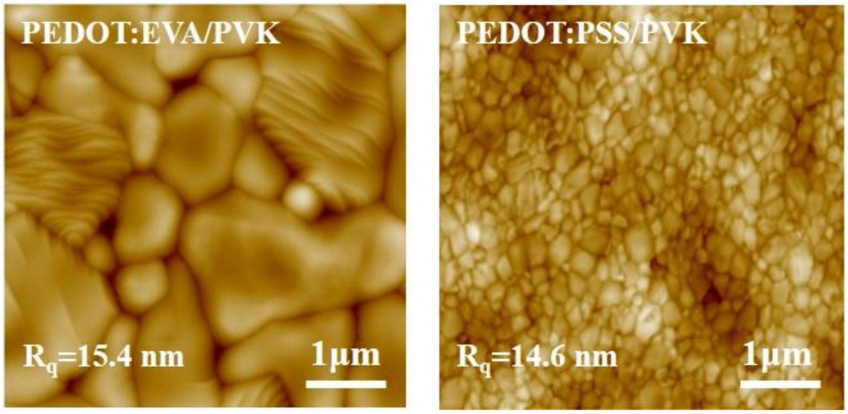

Supplementary Figure 2. Atomic force microscope (AFM) images of perovskite films based on PEDOT:EVA and PEDOT:PSS HTLs.

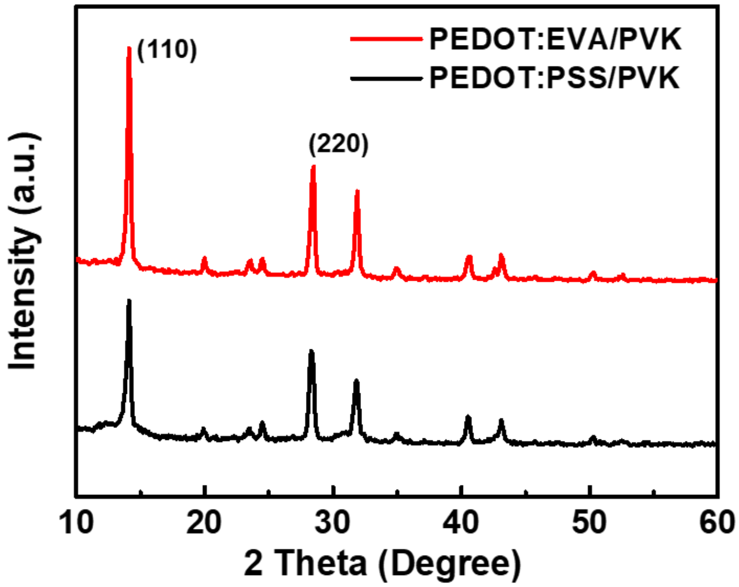

Supplementary Figure 3. X-ray diffraction (XRD) patterns of perovskite films based on PEDOT:EVA and PEDOT:PSS HTLs.

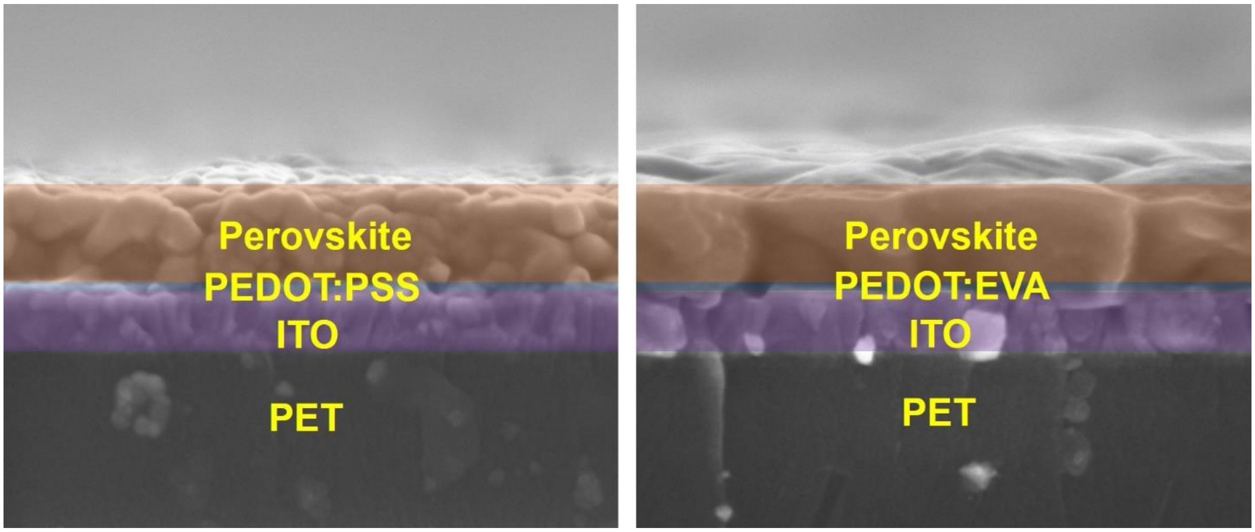

Supplementary Figure 4. The cross-sectional SEM images of perovskite films based on PEDOT: EVA and PEDOT:EVA HTLs.

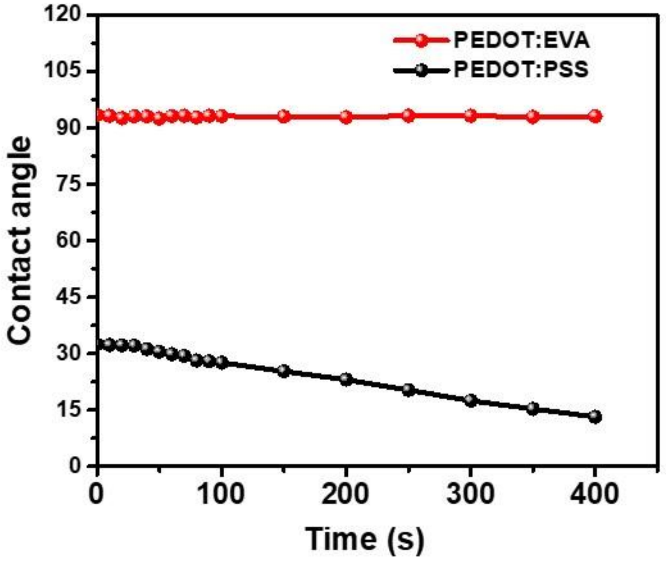

Supplementary Figure 5. Measurement of the contact angle for the PEDOT:EVA and PEDOT:PSS films.

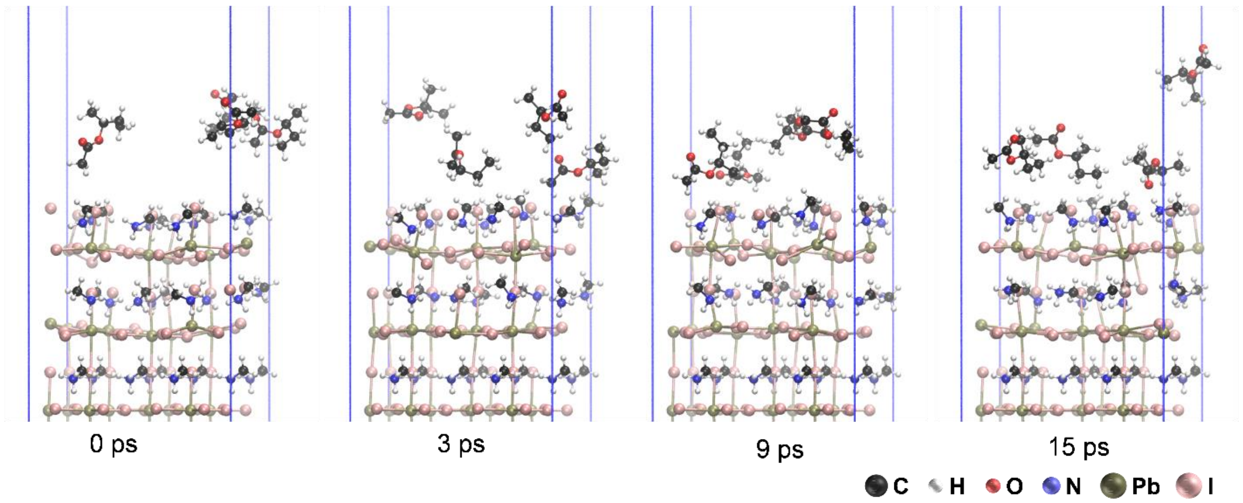

Supplementary Figure 6. The CPMD of MAI-terminated with EVA.

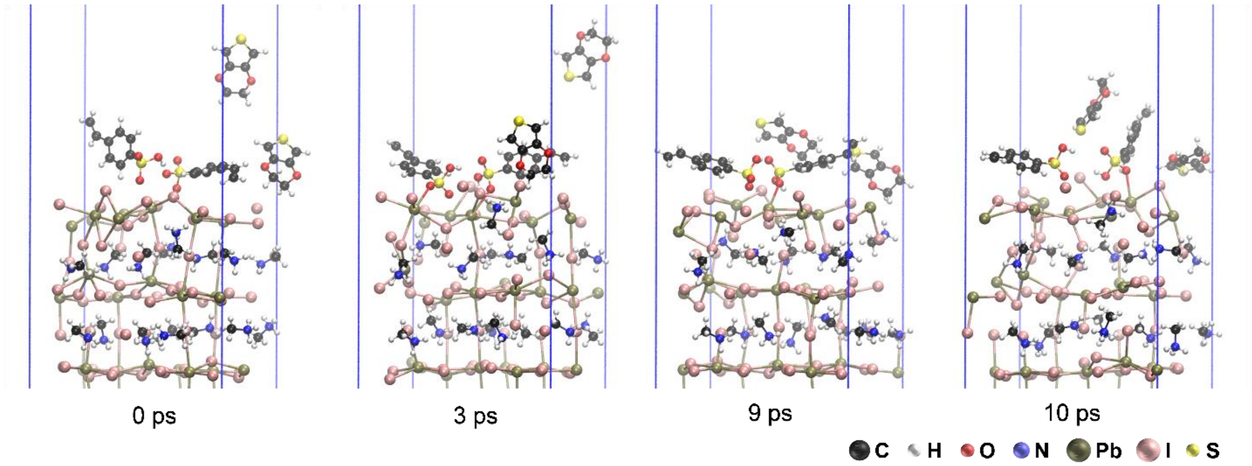

Supplementary Figure 7. The CPMD of PbI_2-terminated with PEDOT:PSS.

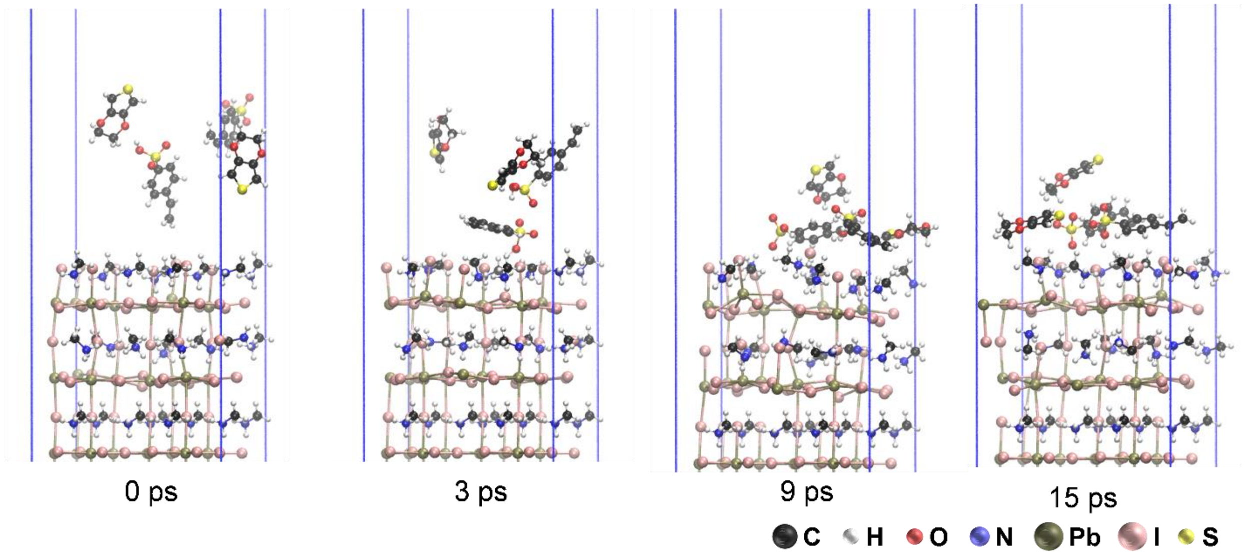

43

44 **Supplementary Figure 8.** The CPMD of MAI-terminated with PEDOT:PSS.

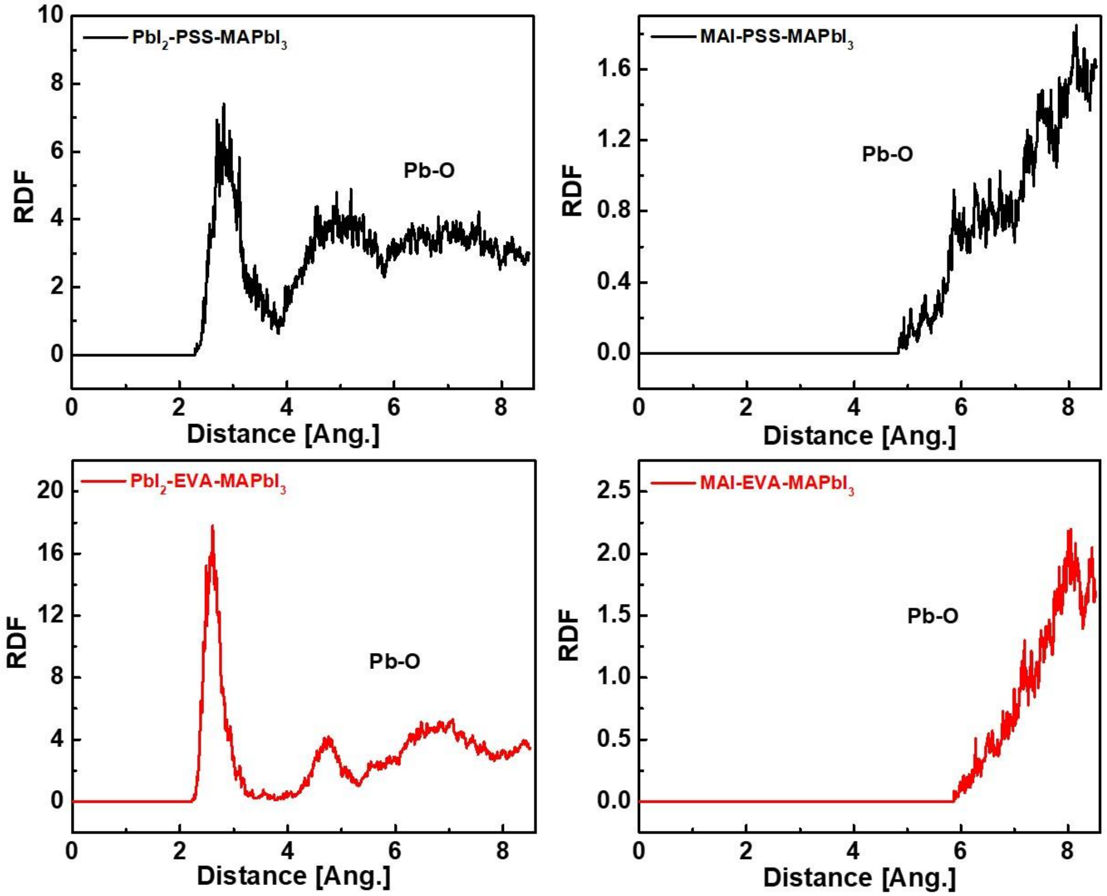

Supplementary Figure 9. Binding energy variation of PbI_2 with EVA or PSS and MAI with EVA or PSS.

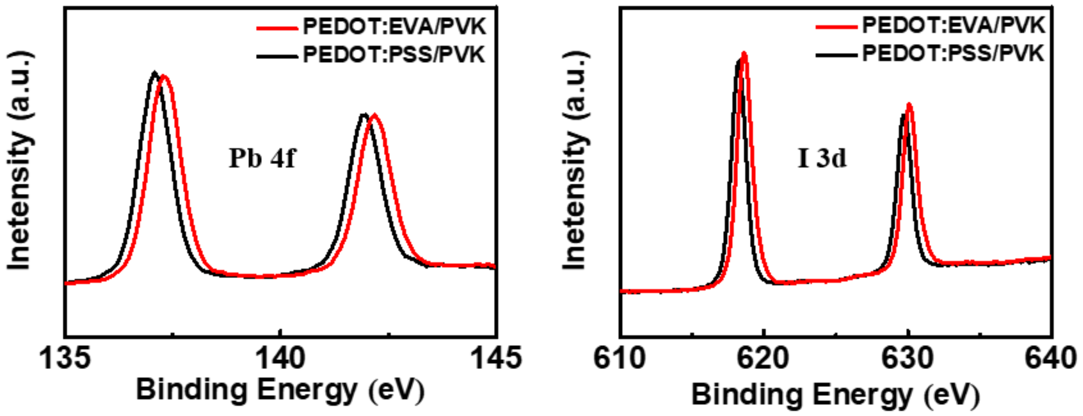

Supplementary Figure 10. XPS spectra depicting the Pb 4f and I 3d peaks of the perovskite films based on PEDOT:EVA and PEDOT:PSS HTLs.

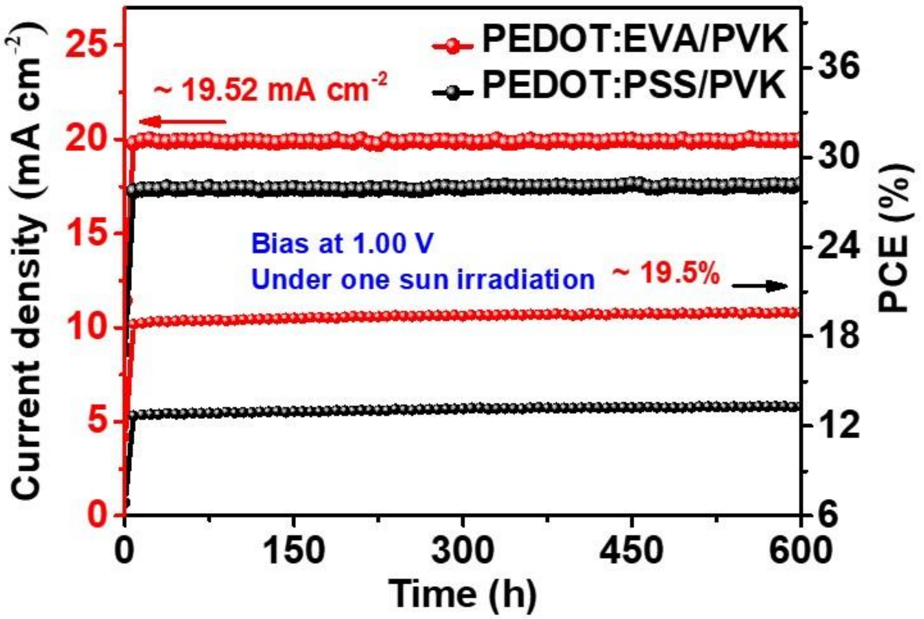

Supplementary Figure 11. Steady-state performance of flexible PSCs based on PEDOT: EVA and PEDOT:PSS.

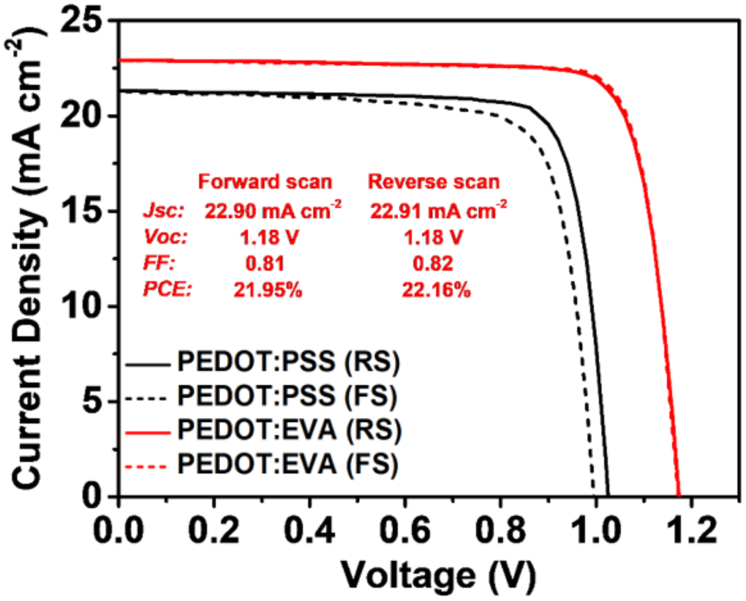

Supplementary Figure 12. *J-V* curves of the PSCs (glass/ITO) based on PEDOT:EVA and PEDOT:PSS layers measured in both the reverse and forward directions.

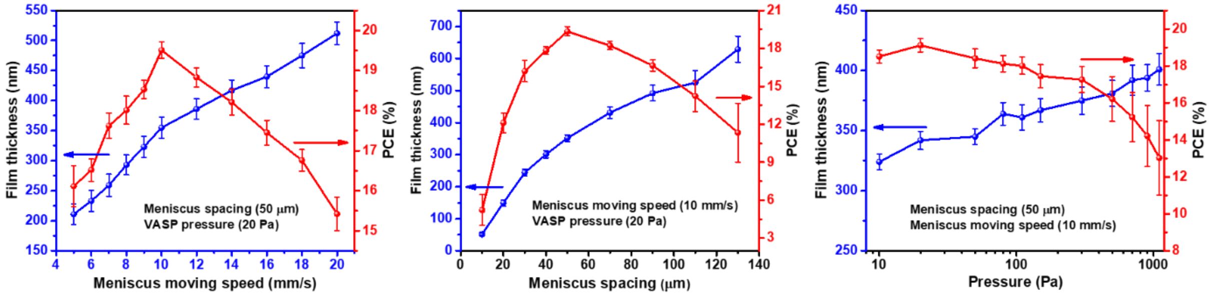

Supplementary Figure 13. The effect of perovskite film thickness based on different meniscus moving speed, meniscus spacing and VASP pressure for the device performance.

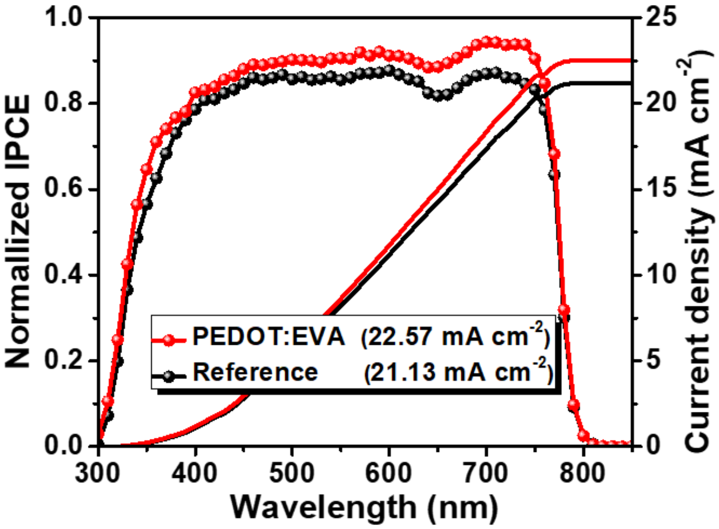

Supplementary Figure 14. The corresponding IPCE spectra of rigid PSCs.

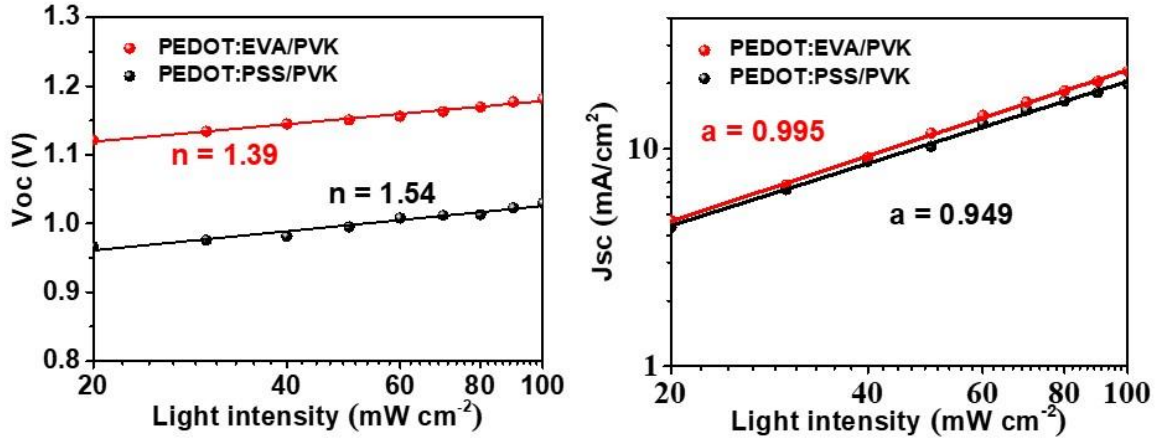

Supplementary Figure 15. V_{oc} and J_{sc} dependence of the PSCs prepared on PEDOT: EVA and PEDOT:PSS substrates at various illumination intensities.

Then, the *J-V* measurement under different illumination intensities is investigated to analyze the charge carrier recombination details (Supplementary Figure 15), the slope of V_{oc} versus illumination intensity is kT/q (where k is the Boltzmann constant, T is the kelvin temperature and q is the elementary charge). The lower slope for the device prepared on PEDOT:EVA substrate (1.39 kT/q compared with 1.54 kT/q of the reference) means that the trap-assisted recombination is effectively suppressed under open-circuit condition. The power law dependence between J_{sc} with illumination intensity can be expressed as $J_{\mathrm{sc}} \propto I^{\alpha}$ (where I is the light intensity and α is the exponential factor), and the higher value of α for the device prepared on PEDOT:EVA substrate (0.995) proves the reduction in bimolecular recombination under short-circuit condition compared with the reference device (0.949).

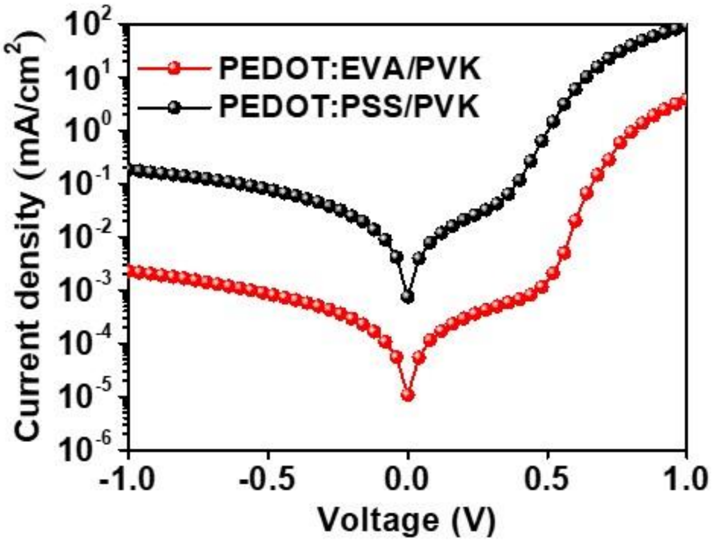

Supplementary Figure 16. Dark *J-V* curves of the PSCs for PEDOT:PSS or PEDOT:EVA substrate.

As shown in Supplementary Figure 16, dark *J-V* curves are characterized to investigate the transport performance of photogenerated carriers, the lower dark current density for the device based on PEDOT:EVA indicates that the more photogenerated carriers can be transmitted through the perovskite films instead of direct shunt, which results in the effective suppression of charge carrier recombination and leakage current. The above results indicate that the PEDOT:EVA HTLs can indeed reduce the trap-assisted and bimolecular recombination, which also means the better perovskite film quality and interfacial ohmic contact.

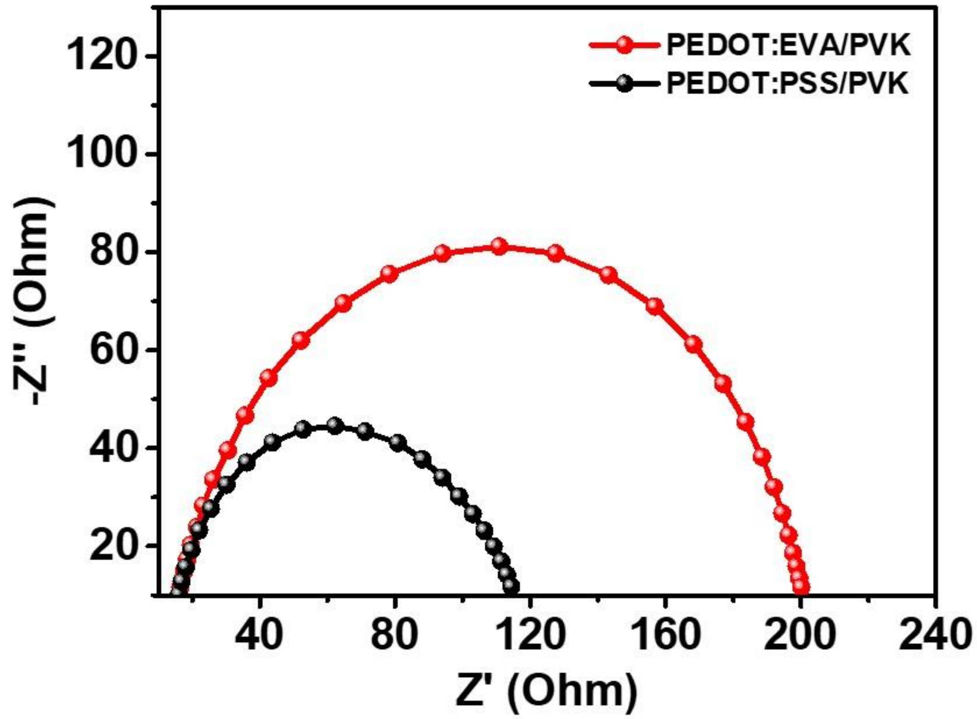

Supplementary Figure 17. The electrical impedance spectroscopy (EIS) data of the ACI devices based on different HTLs.

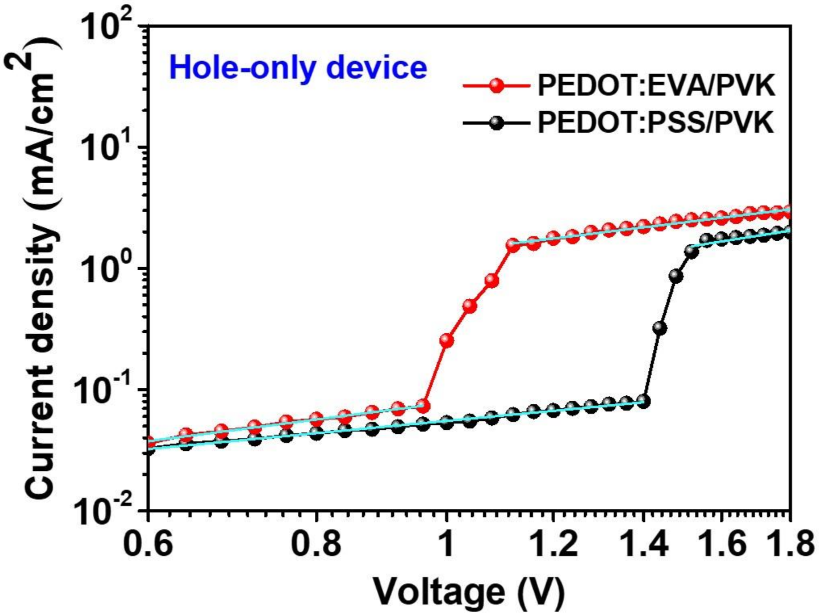

Supplementary Figure 18. The space-charge-limited-current (SCLC) module for perovskite with PEDOT:EVA or PEDOT:PSS hole-only devices.

The PEDOT:EVA HTLs mobility and charge transfer performance are further investigated via the electrical impedance spectroscopy (EIS) and space-charge-limited-current (SCLC) (Supplementary Figure 17 and Supplementary Figure 18), and the measurement results indicate that PEDOT:EVA HTLs can significantly improve the optoelectronic properties of the perovskite films, which is due to the enhanced charge mobility, reduced trap state density and the optimized contact resistance at the charge extraction layer. These improvements are beneficial for more perfect V_{oc} and FF in the PSCs.

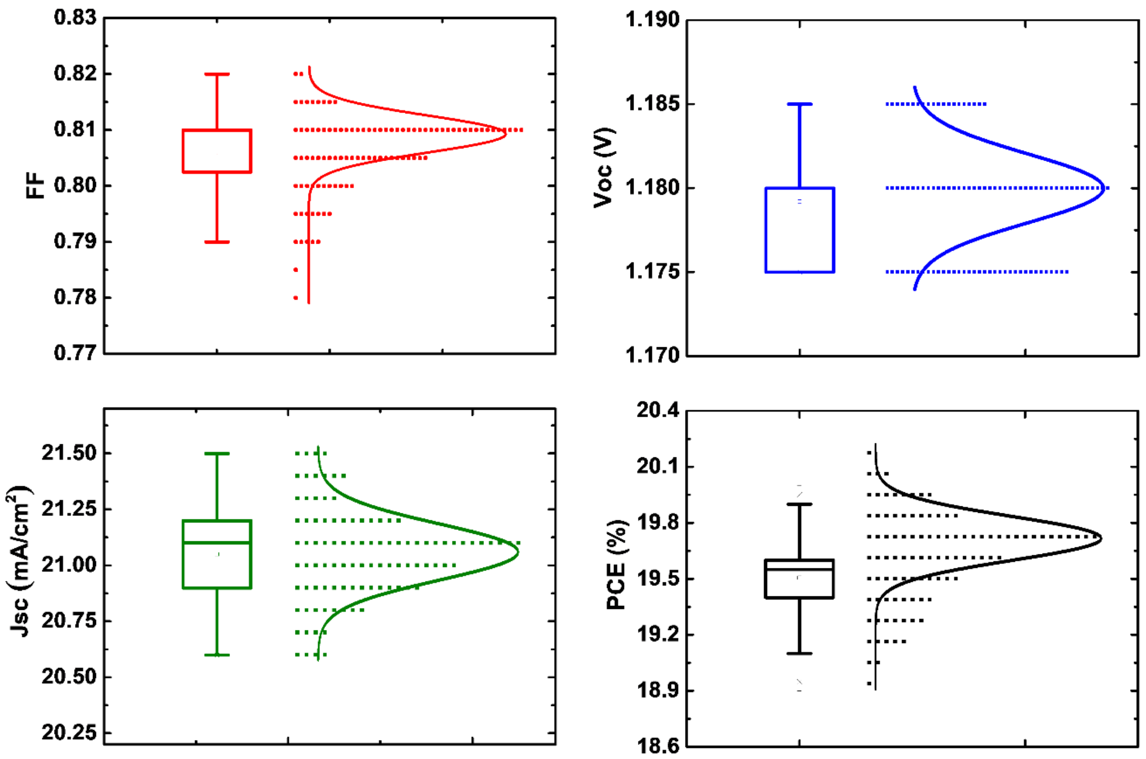

Supplementary Figure 19. The statistics of the flexible PSCs based on PEDOT:EVA.

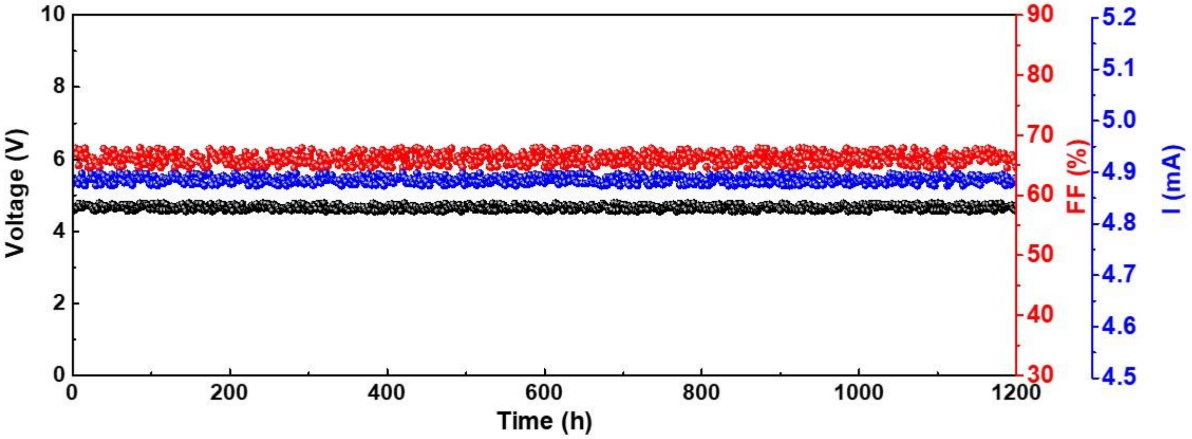

Supplementary Figure 20. Steady-state performance of flexible PSMs based on PEDOT:EVA.

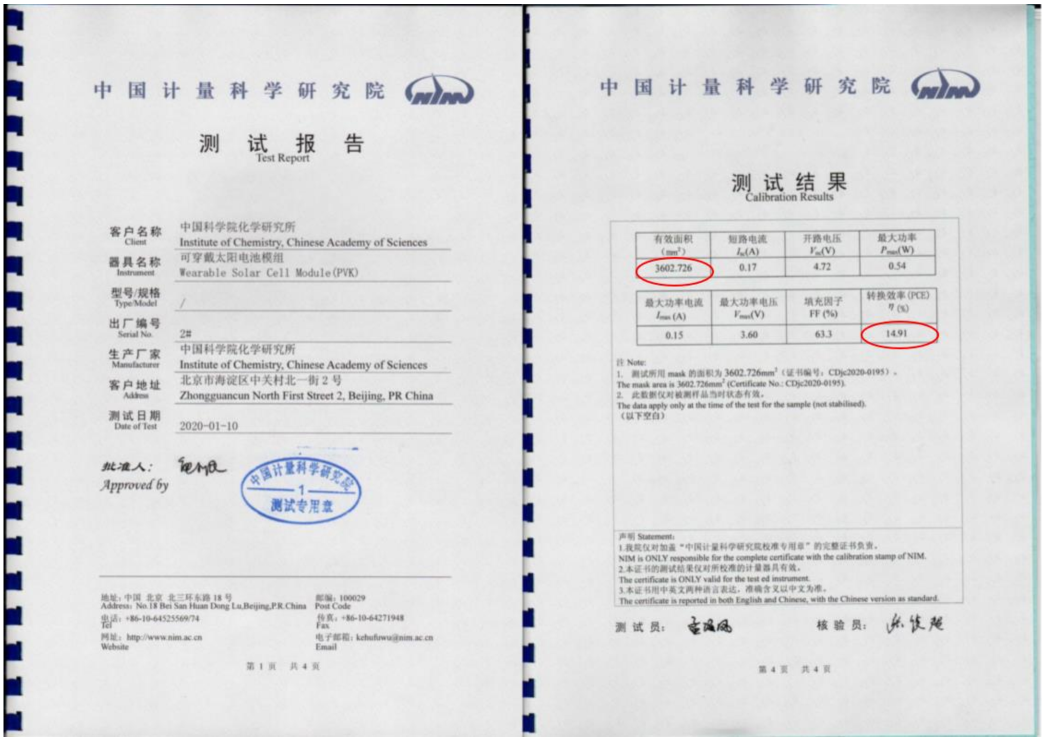

中国计量科学研究院

测 试 报 告
Test Report

客户名称 Client: 中国科学院化学研究所 Institute of Chemistry, Chinese Academy of Sciences
器具名称 Instrument: 可穿戴太阳电池模组 Wearable Solar Cell Module(PVK)
型号/规格 Type/Model: /
出厂编号 Serial No.: 2#
生产厂家 Manufacturer: 中国科学院化学研究所 Institute of Chemistry, Chinese Academy of Sciences
客户地址 Address: 北京市海淀区中关村北一街2号 Zhongguancun North First Street 2, Beijing, PR China
测试日期 Date of Test: 2020-01-10

批准人: Approved by

中国计量科学研究院 1 测试专用章

地址：中国 北京 北三环东路18号 Address: No.18 Bei San Huan Dong Lu,Beijing,P.R.China
邮编：100029 Post Code
电话：+86-10-64525569/74 Tel
传真：+86-10-64271948 Fax
网址：http://www.nim.ac.cn Website
电子邮箱：kehufuwu@nim.ac.cn Email

第 1 页 共 4 页

中国计量科学研究院

测 试 结 果
Calibration Results

有效面积 (mm²)	短路电流 I_{sc}(A)	开路电压 V_{oc}(V)	最大功率 P_{max}(W)
3602.726	0.17	4.72	0.54

最大功率电流 I_{max} (A)	最大功率电压 V_{max}(V)	填充因子 FF (%)	转换效率 (PCE) η (%)
0.15	3.60	63.3	14.91

注 Note:
1. 测试所用 mask 的面积为 3602.726mm²（证书编号：CDjc2020-0195）。
The mask area is 3602.726mm² (Certificate No.: CDjc2020-0195).
2. 此数据仅对被测样品当时状态有效。
The data apply only at the time of the test for the sample (not stabilised).
（以下空白）

声明 Statement:
1.我院仅对加盖"中国计量科学研究院校准专用章"的完整证书负责。
NIM is ONLY responsible for the complete certificate with the calibration stamp of NIM.
2.本证书的测试结果仅对所校准的计量器具有效。
The certificate is ONLY valid for the test ed instrument.
3.本证书用中英文两种语言表达，准确含义以中文为准。
The certificate is reported in both English and Chinese, with the Chinese version as standard.

测试员： 核验员：

第 4 页 共 4 页

Supplementary Figure 21. The device efficiency certification report for perovskite solar cell module with a 36 cm^2 effective area (by National Institute of Metrology, China).

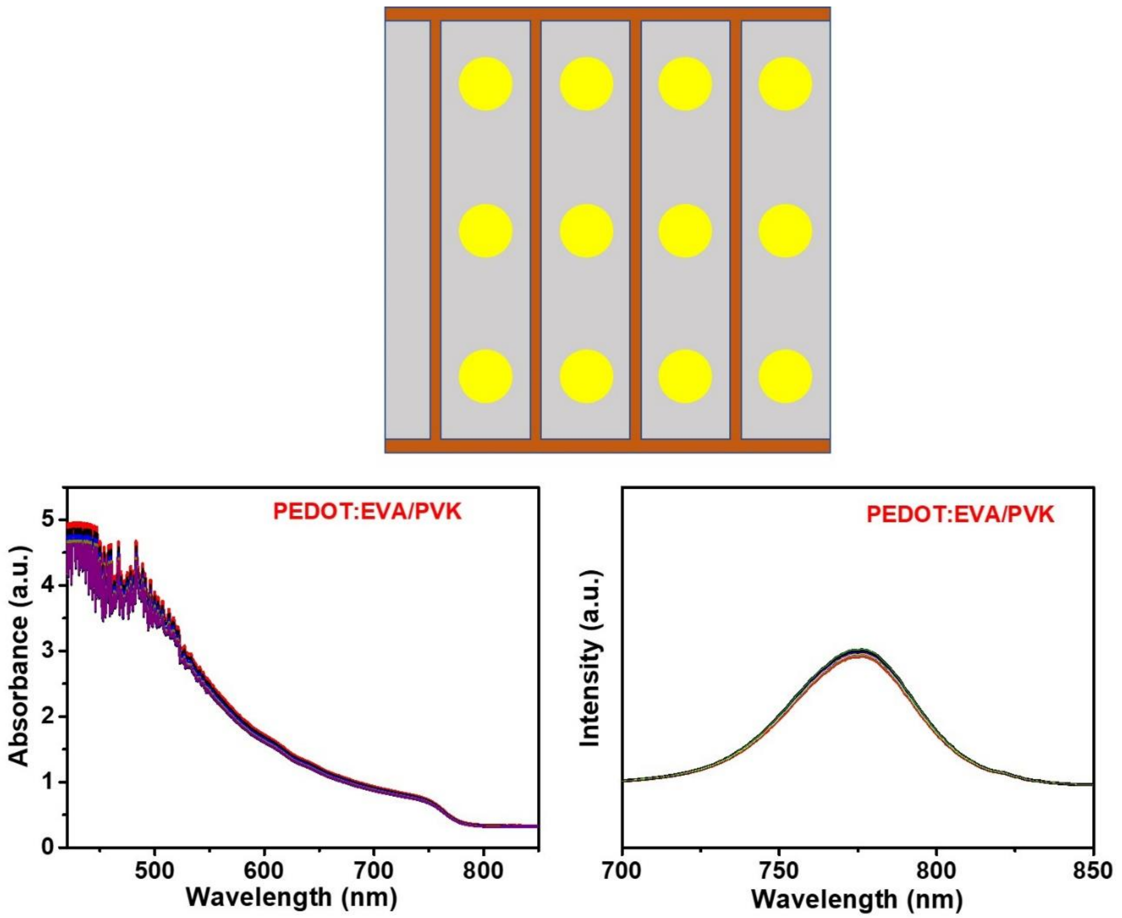

Supplementary Figure 22. Randomly selected 12 pieces from the PSMs are used to investigate the reproducibility. The ultraviolet–visible light absorption spectra and steady state photoluminescence spectra of perovskite films deposited on PEDOT:PSS:EVA of the each piece.

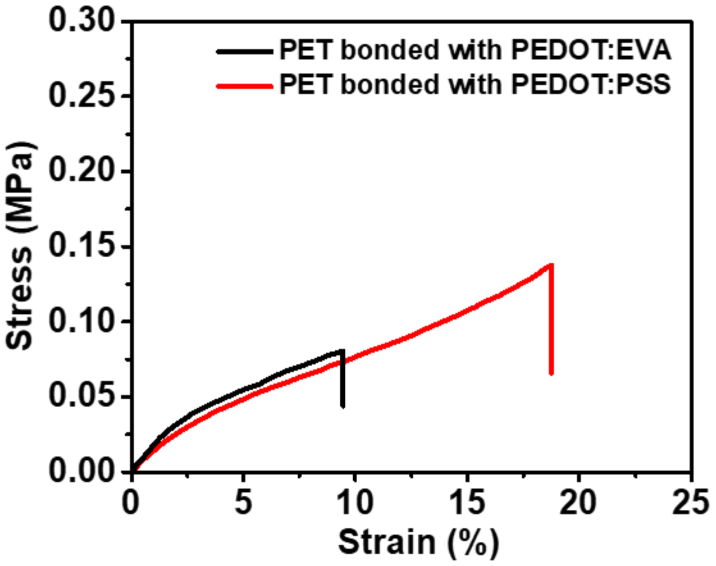

Supplementary Figure 23. The stress-strain curves for the PET material bonded with PEDOT:EVA and PEDOT:PSS.

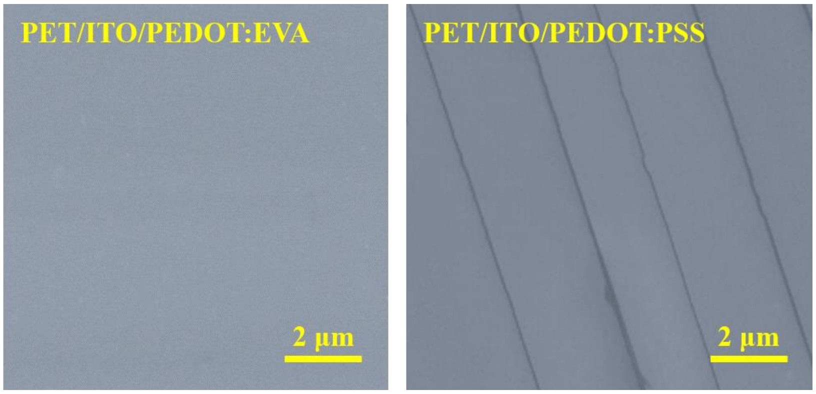

Supplementary Figure 24. The SEM images of PET/ITO/PEDOT:EVA and PET/ITO/PEDOT:PSS bent with 4500 cycles within a curvature radius form flat to 3 mm.

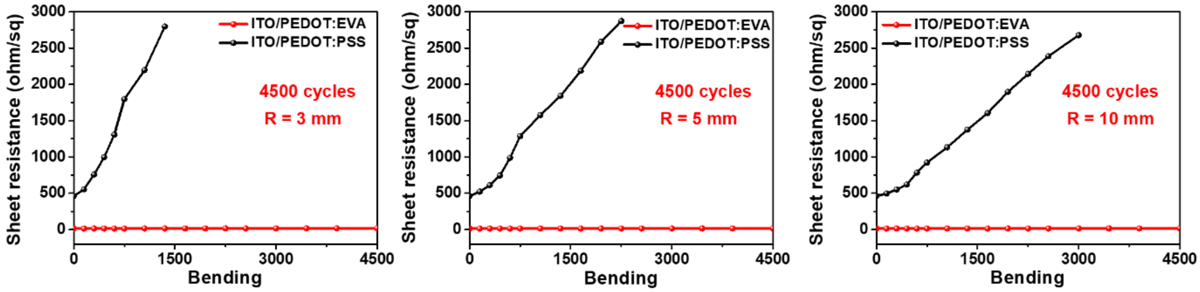

Supplementary Figure 25. Averaged sheet resistance of PET/ITO/PEDOT:EVA and PET/ITO/PEDOT:PSS measured after bending 4500 cycles with a curvature radius.

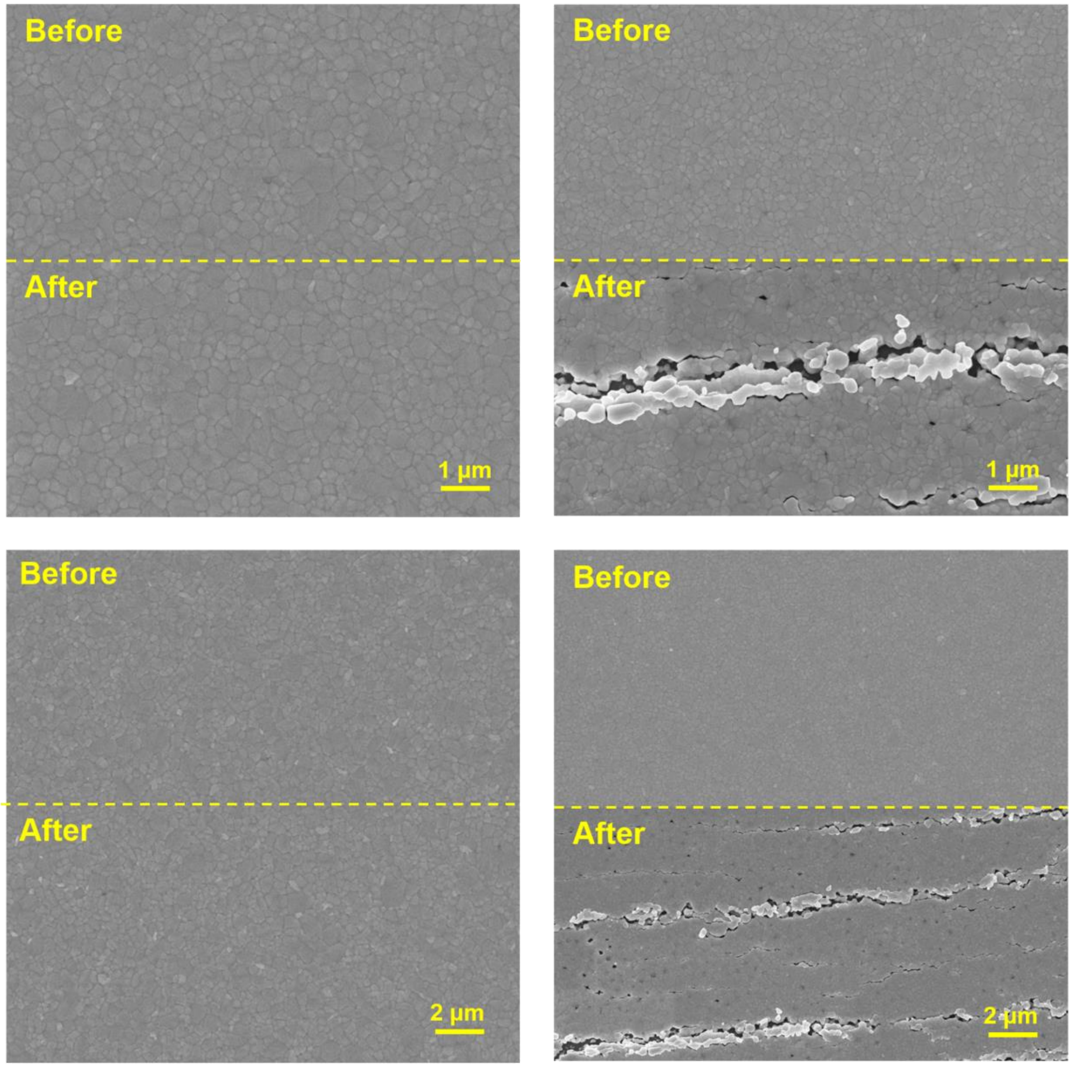

Supplementary Figure 26. The SEM images of flexible PSCs bent with 7000 cycles within a curvature radius form flat to 3 mm.

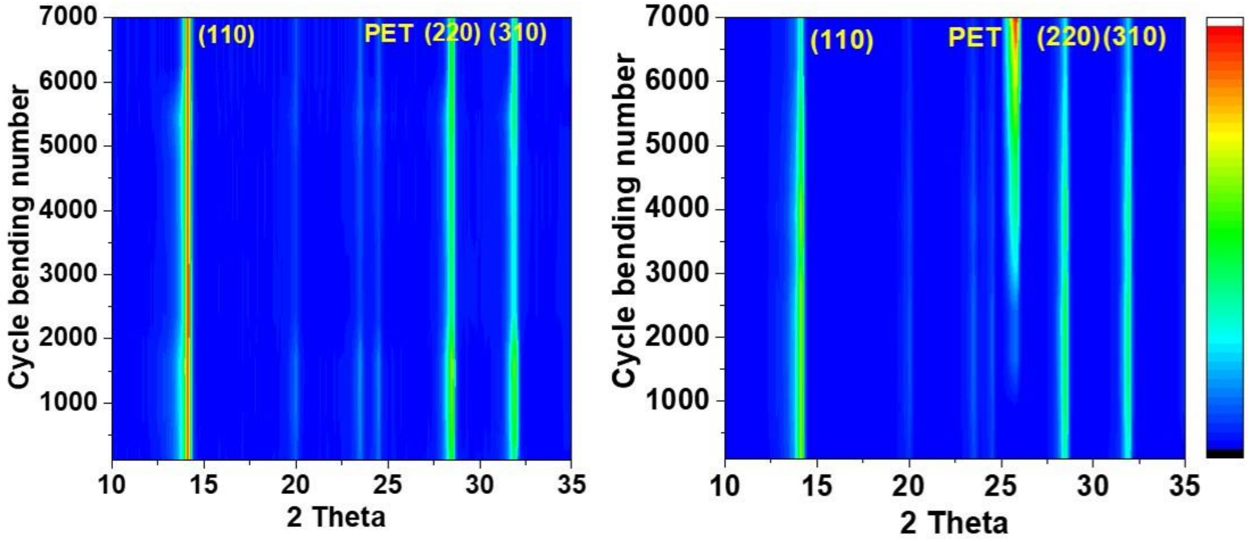

Supplementary Figure 27. XRD spectrum of flexible perovskite films under different cycle bending times prepared on PEDOT:EVA and PEDOT:PSS.

The X-coordinate of XRD pattern in Supplementary Figure 27. is the incident angle, Y-coordinate is the bending times, and the color represents the peak intensity. For the perovskite film prepared on PEDOT:EVA layer, the peak intensity of (110), (220) and (310) did not change significantly after 7000 bending times, which means the structure is not damaged. However, the the peak intensity of (110), (220) and (310) of the perovskite film prepared on the PEDOT:PSS layer has gradually weakened after bending. The characteristic peak of PET at about 26 degree is detected, which proves that the PET is exposed on the surface.

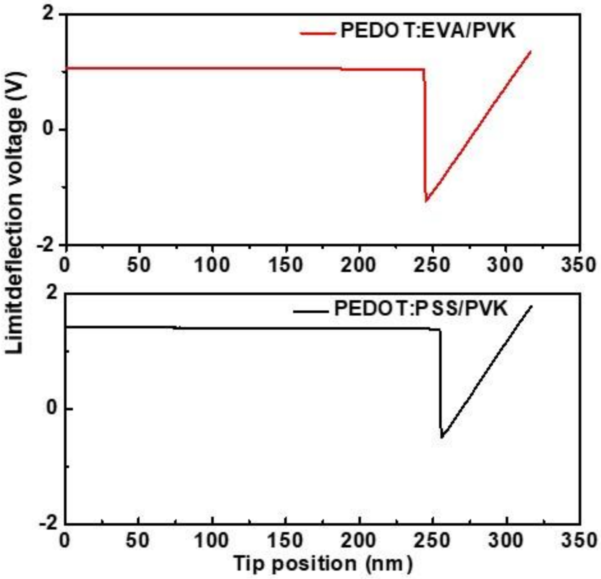

Supplementary Figure 28. Young's Modulus measured by peak-force model of AFM.

Young's Modulus is a common parameter in engineering design for selecting the materials of mechanical parts and is also a physical quantity describing the deformation resistance of solid materials. The Young's Modulus can be regarded as an index to measure the difficulty of elastic deformation. The larger the value of Young's Modulus usually means the greater the stress causing certain elastic deformation, and the more significant rigidity of the material. As for the flexible device, too rigid films will be prone to appear more micro cracks after bending conditions, and the mechanical stability of photoelectric device will be also pessimistic. The introduction of PEDOT:EVA buffer layer not only improves the grain size for the perovskite films, but also reduces the stress accumulation for the flexible films under various bending radius. Therefore, the mechanical stability of flexible device is significantly optimized.

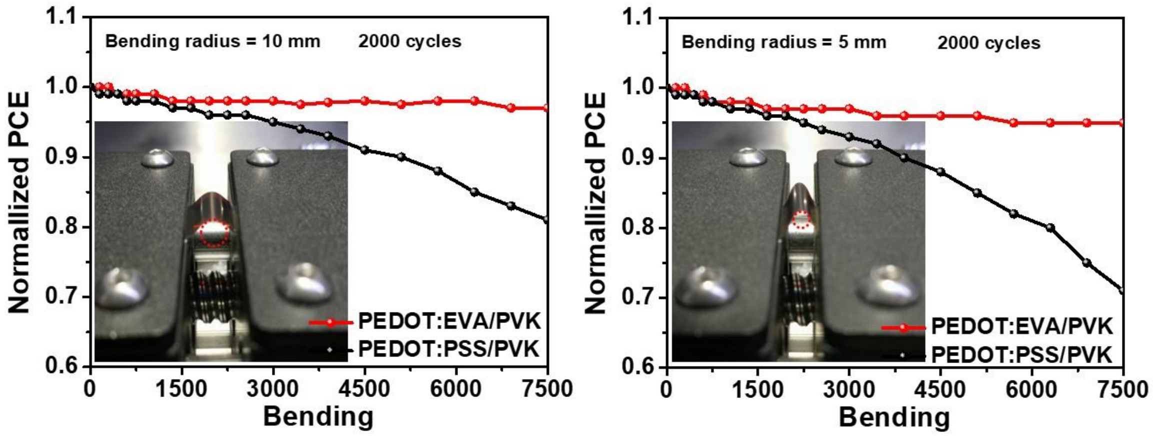

Supplementary Figure 29. Normalized averaged PCE of flexible PSCs chip measured after bending 7000 cycles with a curvature radius.

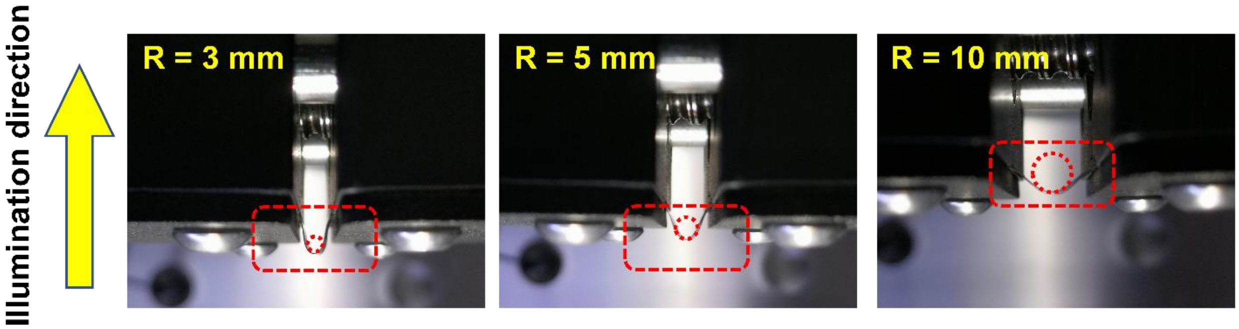

154 **Supplementary Figure 30.** The schematic diagram of bending efficiency measurement.

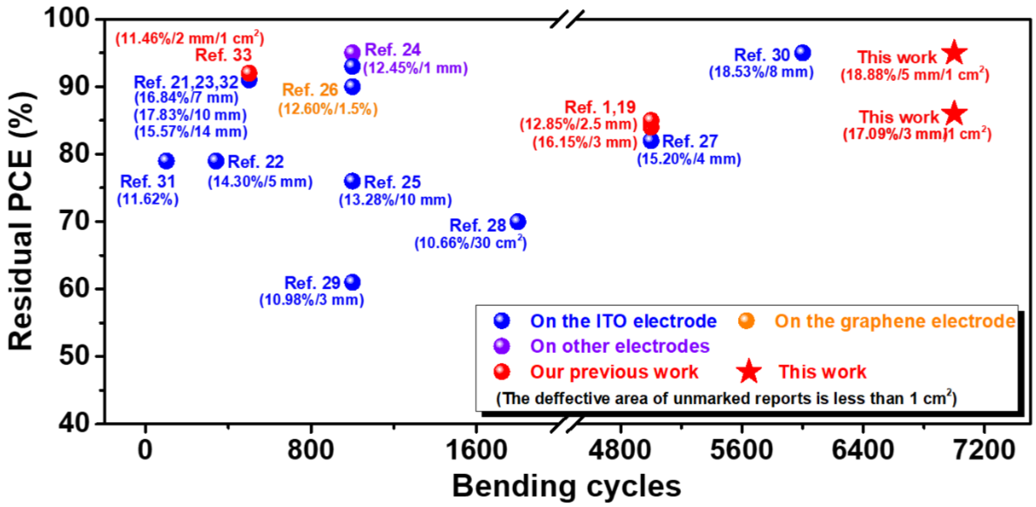

Supplementary Figure 31. The summary of residual PCE for the recently reported flexible PSCs after bending cycles. The blue dots, orange dots and purple dots represent the reports based on ITO electrode, graphene electrode and other electrodes, respectively. The red dots represent our previous work and the red solid stars represent this work

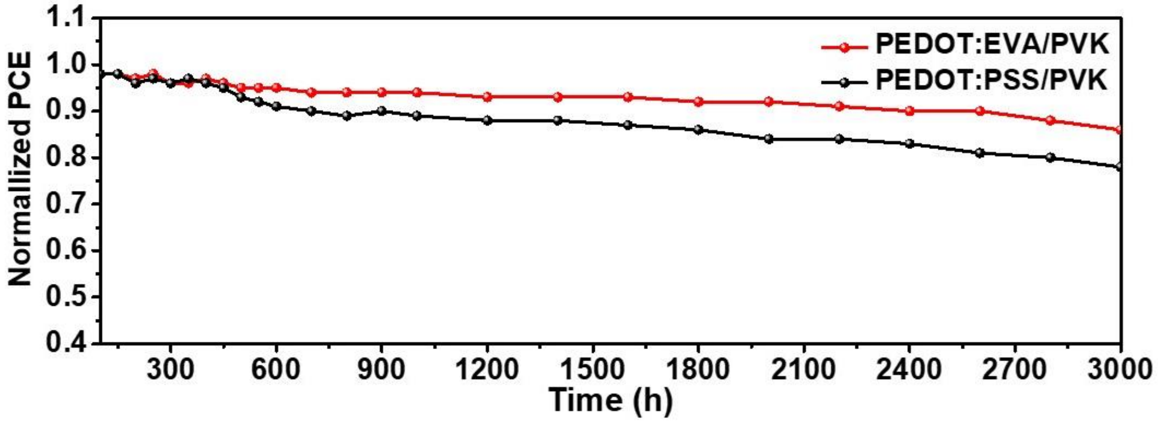

Supplementary Figure 32. Normalized PCE of encapsulated PSCs based on different electrodes.

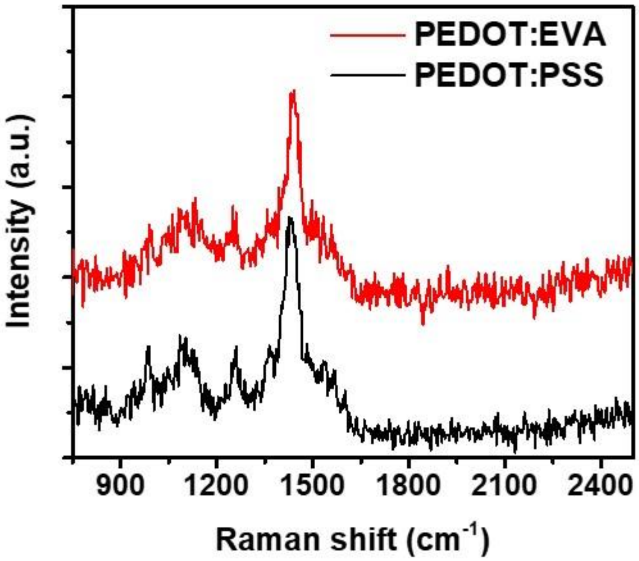

162

163 **Supplementary Figure 33.** Raman spectra of PEDOT:EVA and PEDOT:PSS.

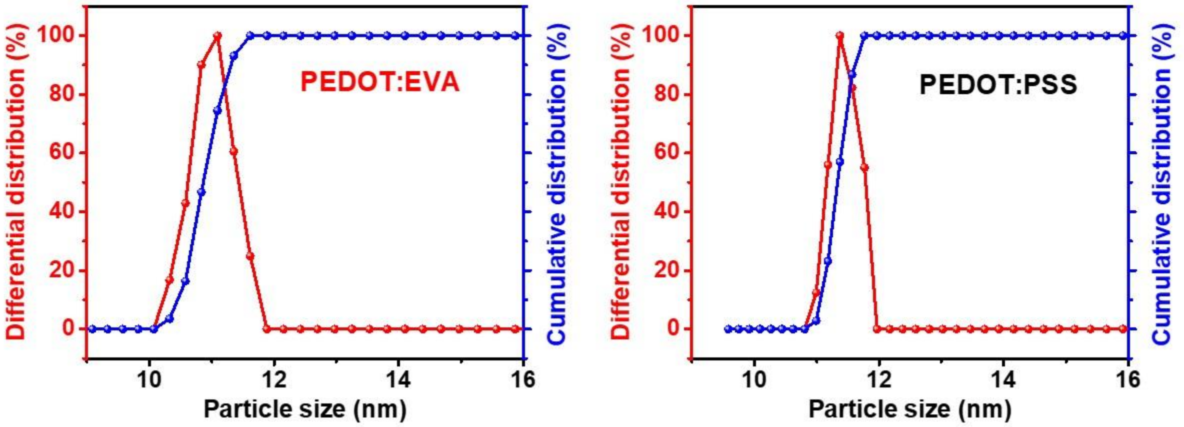

Supplementary Figure 34. Particle size distribution of PEDOT:EVA and PEDOT:PSS solution.

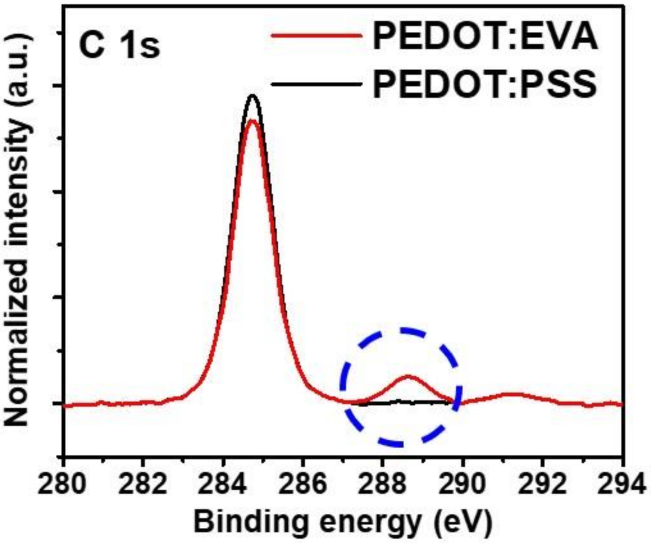

Supplementary Figure 35. XPS spectra depicting the C 1s peaks of PEDOT:EVA and PEDOT:PSS films.

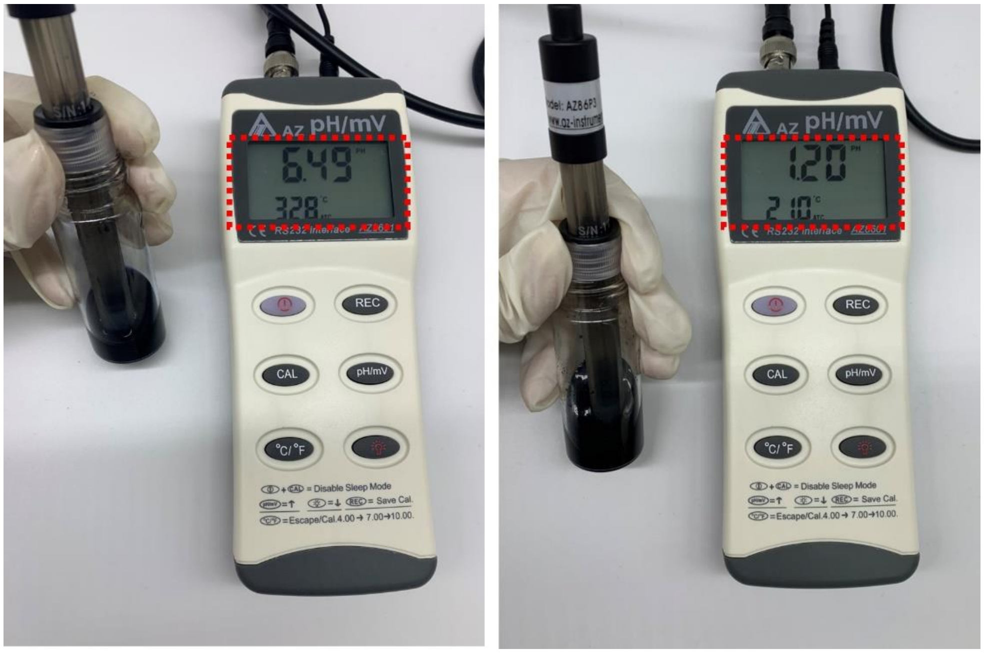

Supplementary Figure 36. The acid base (pH value) of PEDOT:EVA and PEDOT:PSS ink.

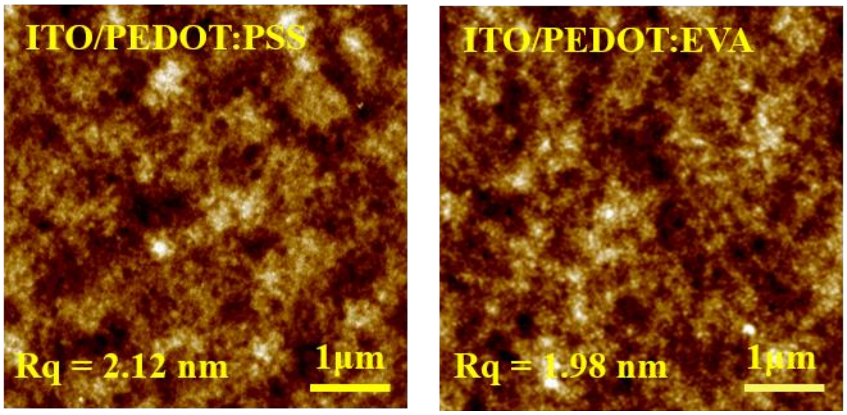

Supplementary Figure 37. Atomic force microscope (AFM) images of ITO/PEDOT and ITO/PEDOT:EVA films.

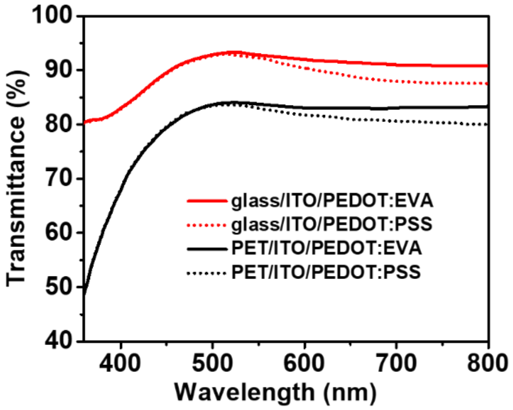

Supplementary Figure 38. Transmission spectra of glass/ITO/PEDOT:EVA, glass/ITO/PEDOT:PSS, PET/ITO/PEDOT:EVA and PET/ITO/PEDOT:PSS.

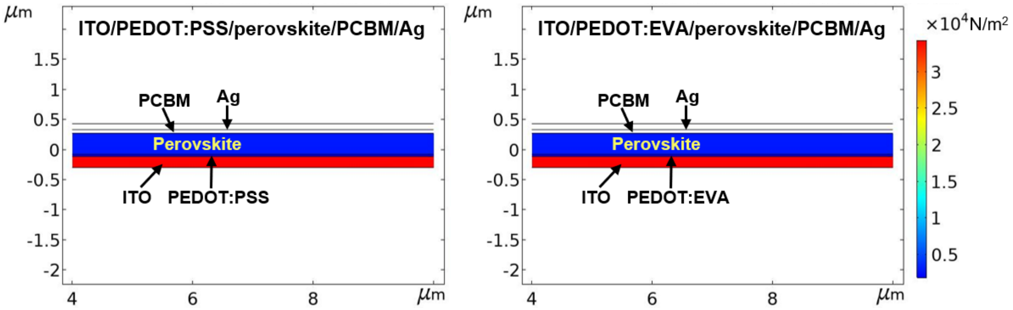

Supplementary Figure 39. Finite-elements simulation model of flexible PSCs upon PEDOT:EVA and PEDOT:PSS.

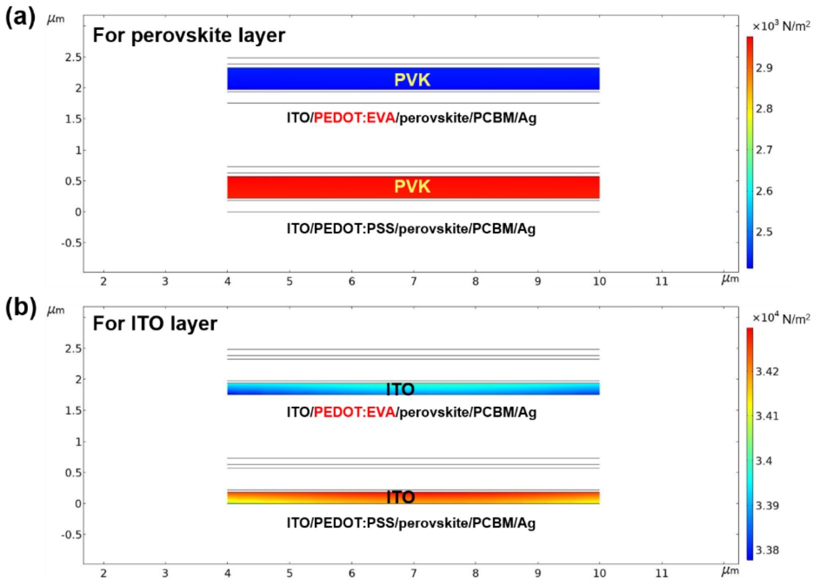

Supplementary Figure 40. Finite-elements simulation for the perovskite and ITO layers upon PEDOT:EVA and PEDOT:PSS.

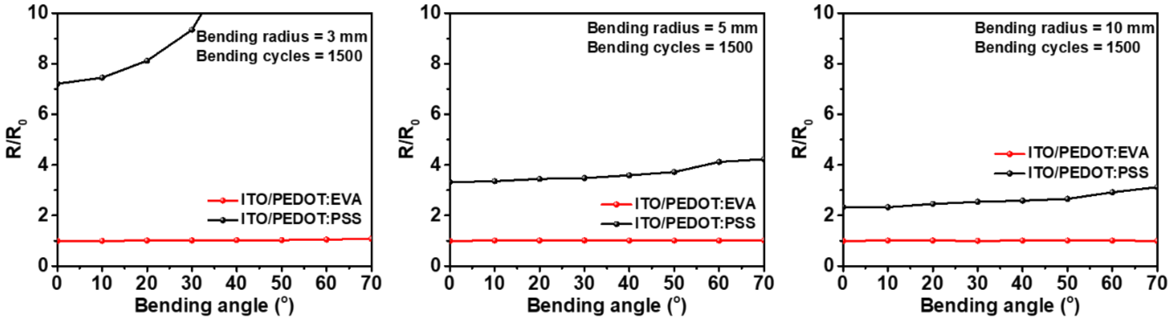

Supplementary Figure 41. The conductivity of ITO film coated with PEDOT:EVA and PEDOT:PSS under different bending angles after 1500 cycles.

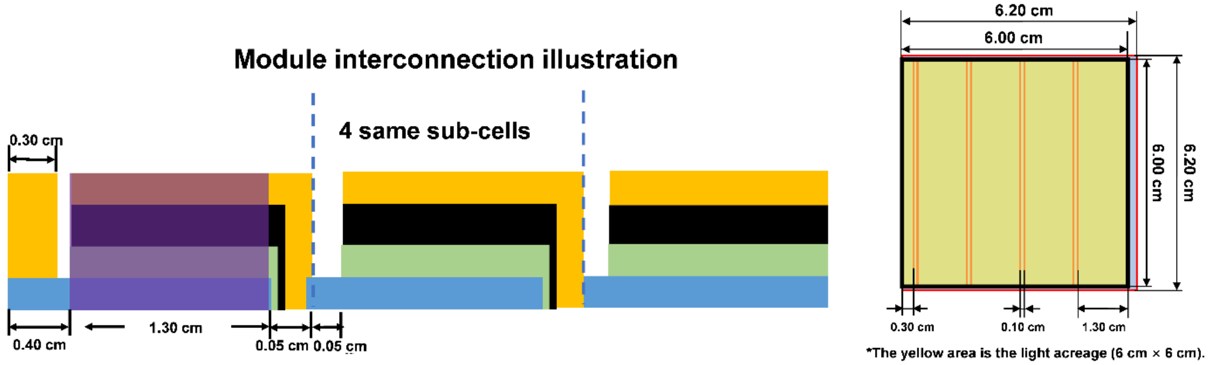

Supplementary Figure 42. The structure of perovskite solar modules.

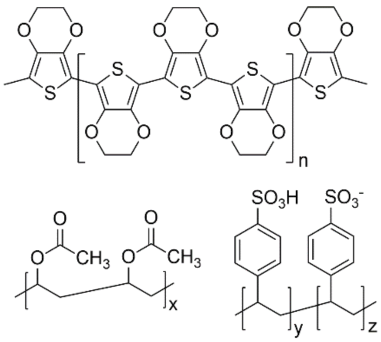

Supplementary Figure 43. The conductivity of ITO film coated with PEDOT:EVA and PEDOT:PSS under different bending angles after 1500 cycles.

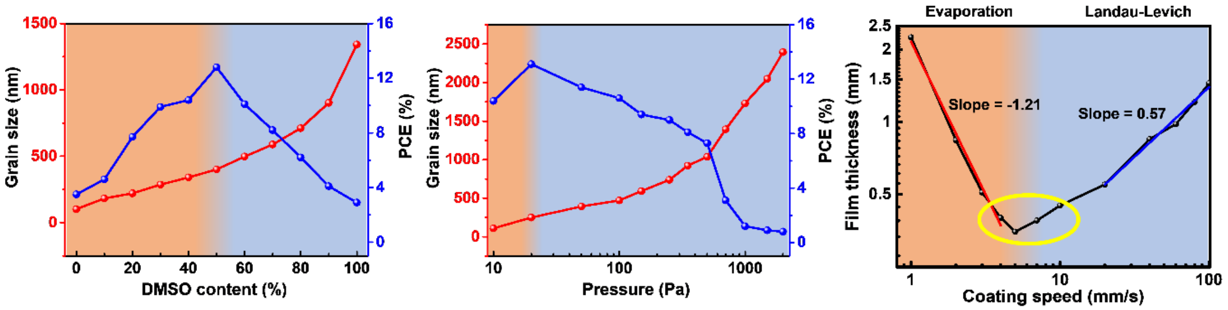

Supplementary Figure 44. The statistical diagram of the relation between specific parameters of meniscus-coating with grain size and device efficiency.

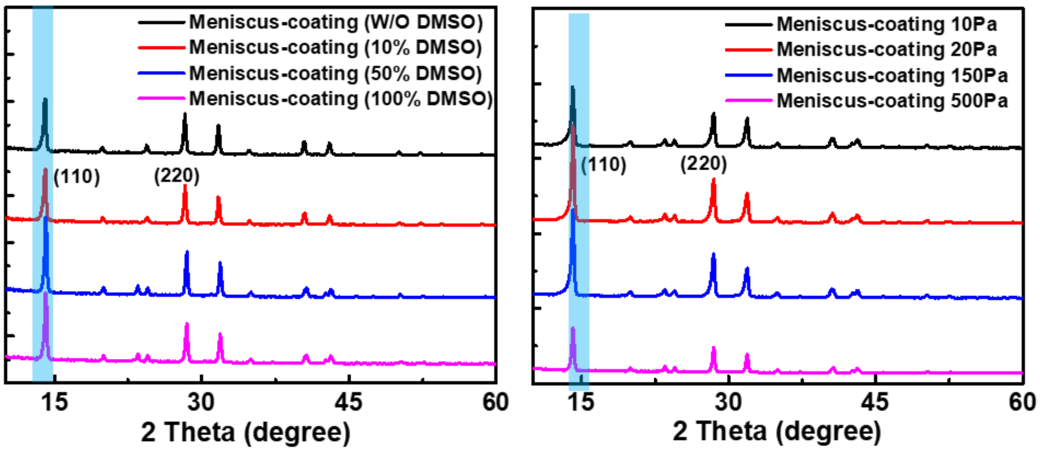

Supplementary Figure 45. X-ray diffraction (XRD) patterns of perovskite films prepared with different meniscus-coating parameters.

Supplementary Table 1. Water contact angle value of PEDOT:EVA and PEDOT:PSS films.

Time (s)	PEDOT:EVA	PEDOT:PSS
0	93.4	32.5
10	93.2	32.3
20	92.6	32.2
30	93.1	32.1
40	93.0	31.2
50	92.5	30.5
60	93.1	29.8
70	93.2	29.4
80	92.8	28.2
90	93.2	28.0
100	93.1	27.6
150	93.0	25.3
200	92.8	23.1
250	93.2	20.3
300	93.2	17.5
350	92.9	15.3
400	93.1	13.2

Supplementary Table 2. The carrier recombination lifetime.

HTLs	Fast phase lifetime (τ_1)	Slow phase lifetime (τ_2)
PEDOT:EVA	9.8 ns	544.1 ns
PEDOT:PSS	14.6 ns	994.5 ns

Supplementary Table 3. Photovoltaic performance of the PSCs based on different rigid substrates.

**Device**[a)]		J_{sc} **(mA cm^{-2})**	V_{oc} **(V)**	**FF**	**PCE (%)**
Reference	Reverse	21.32	1.02	0.81	17.61
	Forward	21.31	1.00	0.77	16.34
	Average	21.12 ± 0.44	1.01 ± 0.01	0.77 ± 0.03	16.43 ± 1.02
PEDOT:EVA	Reverse	22.91	1.18	0.82	22.16
	Forward	22.90	1.18	0.81	21.95
	Average	22.84 ± 0.21	1.18 ± 0.01	0.80 ± 0.01	21.56 ± 0.45

The average and standard deviation values are based on 50 cells and the “±” is defined as the error bar. [a)] The effective area for devices is 1.01 cm^2. The average and standard deviation values are based on 50 cells.

Supplementary Table 4. The effect of meniscus-coating parameters for the device performance.

Constant	Moving speed (mm s^{-1})	Film thickness (nm)	PCE (%)
	5	211 ± 17.1	16.11 ± 0.51
	6	233 ± 17.7	16.52 ± 0.28
	7	259 ± 18.4	17.62 ± 0.32
	8	293 ± 16.7	18.01 ± 0.35
**Meniscus spacing**	9	323 ± 17.3	18.53 ± 0.23
**(50 mm)**	10	355 ± 17.5	19.51 ± 0.21
**VASP pressure (20 Pa)**	12	386 ± 16.8	18.83 ± 0.24
	14	417 ± 17.2	18.21 ± 0.32
	16	440 ± 18.3	17.45 ± 0.31
	18	475 ± 20.1	16.76 ± 0.27
	20	512 ± 18.6	15.42 ± 0.42
**Constant**	**Moving spacing (μm)**	**Film thickness (nm)**	**PCE (%)**
	10	52 ± 5.2	5.22 ± 1.21
	20	150 ± 9.4	12.12 ± 0.79
	30	244 ± 10.3	16.21 ± 0.85
**Meniscus moving speed**	40	301 ± 11.3	17.83 ± 0.29
**(10 mm s^{-1})**	50	352 ± 10.6	19.35 ± 0.37
**VASP pressure (20 Pa)**	70	431 ± 17.3	18.23 ± 0.33
	90	492 ± 24.5	16.65 ± 0.44
	110	525 ± 36.4	14.23 ± 1.21
	130	629 ± 40.2	11.34 ± 2.32
**Constant**	**Pressure (Pa)**	**Film thickness (nm)**	**PCE (%)**
	10	324 ± 6.7	18.51 ± 0.34
	20	342 ± 7.6	19.12 ± 0.36
	50	345 ± 6.5	18.41 ± 0.51
**Meniscus spacing (50**	80	364 ± 9.4	18.12 ± 0.44
**mm)**	110	361 ± 10.5	18.01 ± 0.46
**Meniscus moving speed**	150	367 ± 9.7	17.45 ± 0.62
**(10 mm s^{-1})**	300	375 ± 11.4	17.26 ± 0.71
	500	381 ± 10.9	16.21 ± 1.22
	700	392 ± 12.6	15.23 ± 1.34
	900	394 ± 11.0	14.21 ± 1.65
	1100	401 ± 13.1	13.02 ± 2.01

Supplementary Table 5. The mobility of hole-only devices for the PSCs based on different substrates.

**Sample**	**Hole mobility ($cm^2V^{-1}s^{-1}$)**
PEDOT:PSS	9.35×10^{-5}
PEDOT:EVA	1.19×10^{-4}

Supplementary Table 6. Photovoltaic parameters of the flexible PSCs (1.01 cm^2) based on PEDOT:EVA HTLs.

**Sample No.**	V_{oc} (V)	J_{sc} (mA cm^{-2})	FF	PCE (%)	**Sample No.**	V_{oc} (V)	J_{sc} (mA cm^{-2})	FF	PCE (%)
**1**	1.18	20.53	0.80	19.38	**26**	1.18	21.02	0.80	19.84
**2**	1.18	21.20	0.79	19.76	**27**	1.18	20.86	0.80	19.69
**3**	1.17	21.14	0.80	19.78	**28**	1.16	20.17	0.82	19.18
**4**	1.18	20.31	0.82	19.65	**29**	1.17	20.45	0.80	19.14
**5**	1.17	20.68	0.80	19.35	**30**	1.18	20.41	0.79	19.02
**6**	1.17	21.10	0.79	19.50	**31**	1.18	20.44	0.81	19.53
**7**	1.17	20.71	0.82	19.86	**32**	1.18	20.32	0.81	19.42
**8**	1.18	20.77	0.81	19.85	**33**	1.18	20.59	0.81	19.67
**9**	1.18	20.65	0.81	19.73	**34**	1.18	21.11	0.80	19.91
**10**	1.18	20.44	0.81	19.53	**35**	1.17	21.01	0.80	19.66
**11**	1.18	20.73	0.80	19.56	**36**	1.17	21.44	0.78	19.56
**12**	1.17	20.48	0.80	19.16	**37**	1.17	21.12	0.77	19.02
**13**	1.18	20.65	0.81	19.73	**38**	1.18	20.54	0.82	19.87
**14**	1.18	20.78	0.81	19.86	**39**	1.17	20.26	0.81	19.20
**15**	1.18	20.27	0.80	19.13	**40**	1.18	20.61	0.79	19.20
**16**	1.18	20.24	0.81	19.34	**41**	1.18	20.98	0.80	19.80
**17**	1.16	20.86	0.80	19.35	**42**	1.17	20.19	0.81	19.13
**18**	1.17	20.68	0.82	19.84	**43**	1.18	20.32	0.81	19.42
**19**	1.17	21.06	0.79	19.46	**44**	1.15	20.76	0.81	19.33
**20**	1.18	21.21	0.77	19.27	**45**	1.18	21.24	0.79	19.79
**21**	1.17	20.60	0.79	19.04	**46**	1.17	20.78	0.81	19.69
**22**	1.18	20.72	0.79	19.29	**47**	1.18	20.35	0.81	19.45
**23**	1.16	20.99	0.79	19.23	**48**	1.17	21.14	0.78	19.29
**24**	1.18	21.01	0.80	19.83	**49**	1.18	21.12	0.80	19.82
**25**	1.18	21.13	0.80	19.91	**50**	1.18	20.21	0.79	18.83

Each kind of devices are from five batches counting 50 cells in total.

Supplementary Table 7. Photovoltaic parameters of the flexible PSCs (36.00 cm^2) based on PEDOT:EVA HTLs.

Sample No.	V_{oc} (V)	J_{sc} (mA cm^{-2})	FF	PCE (%)	Sample No.	V_{oc} (V)	J_{sc} (mA cm^{-2})	FF	PCE (%)
**1**	4.70	4.83	0.65	14.75	**26**	4.70	4.87	0.62	14.19
**2**	4.71	4.93	0.65	15.09	**27**	4.71	4.89	0.66	15.20
**3**	4.67	4.93	0.68	15.65	**28**	4.73	4.83	0.64	14.62
**4**	4.70	4.79	0.70	15.75	**29**	4.71	4.88	0.63	14.48
**5**	4.70	4.91	0.61	14.07	**30**	4.67	4.88	0.65	14.81
**6**	4.72	4.78	0.62	13.98	**31**	4.69	4.79	0.64	14.37
**7**	4.70	4.83	0.67	15.20	**32**	4.69	4.91	0.64	14.73
**8**	4.72	4.92	0.62	14.39	**33**	4.67	4.79	0.66	14.76
**9**	4.68	4.81	0.63	14.18	**34**	4.69	4.87	0.65	14.84
**10**	4.73	4.91	0.61	14.16	**35**	4.68	4.89	0.66	15.10
**11**	4.73	4.85	0.68	15.59	**36**	4.71	4.81	0.66	14.95
**12**	4.68	4.91	0.63	14.47	**37**	4.70	4.79	0.63	14.18
**13**	4.70	4.77	0.67	15.02	**38**	4.71	4.91	0.61	14.10
**14**	4.70	4.93	0.63	14.59	**39**	4.69	4.88	0.66	15.10
**15**	4.67	4.91	0.64	14.67	**40**	4.70	4.81	0.66	14.92
**16**	4.69	4.91	0.66	15.19	**41**	4.73	4.83	0.66	15.07
**17**	4.73	4.84	0.66	15.10	**42**	4.70	4.88	0.64	14.67
**18**	4.71	4.93	0.63	14.62	**43**	4.68	4.94	0.65	15.02
**19**	4.68	4.93	0.65	14.99	**44**	4.68	4.89	0.65	14.87
**20**	4.68	4.81	0.65	14.63	**45**	4.71	4.84	0.64	14.58
**21**	4.70	4.93	0.67	15.52	**46**	4.70	4.90	0.63	14.50
**22**	4.69	4.94	0.63	14.59	**47**	4.73	4.78	0.61	13.79
**23**	4.71	4.87	0.65	14.90	**48**	4.66	4.94	0.64	14.73
**24**	4.68	4.83	0.65	14.69	**49**	4.67	4.78	0.63	14.06
**25**	4.73	4.91	0.64	14.86	**50**	4.69	4.80	0.65	14.63

Each kind of devices are from five batches counting 50 cells in total.

Supplementary Table 8. Mechanical properties of flexible ITO/HTLs.

Materials	Thickness (nm)	Young's modules (Mpa)	Density (ρ, g cm^{-3})	Poisson's ratio
ITO/PEDOT:EVA	45	139	1.34	0.25
ITO/PEDOT:PSS	45	258	1.41	0.36

Young's Modulus is measured by the peak force mode of atomic force microscope.

Supplementary Table 9. Devices performance of flexible PSCs with different bending angle.

Test condition	PCE	Integration PCE
Bending angle (0 degree)	19.86	-
Bending angle (10 degree)	19.51	19.82
Bending angle (20 degree)	18.57	19.76
Bending angle (30 degree)	16.83	19.43
Bending angle (40 degree)	14.36	18.75
Bending angle (50 degree)	12.08	18.79
Bending angle (60 degree)	9.31	18.62
Bending angle (70 degree)	6.30	18.41
Bending angle (80 degree)	0.53	3.06

Supplementary Table 10. Summary of residual PCE for the recently reported flexible PSCs.

Structure	PCE (%)	Bending cycles/ bending radius	Residual PCE (%)	Journal
PET/ITO/E-SnO_2/PVK/Spiro-OMeTAD/Au	18.28	500 cycles/7 mm	16.84	Nat. Commun.[21]
PET/ITO/SnO_2/PVK/C_{60}-SAM/PVK/ Spiro-OMeTAD/Au	17.96	340 cycles/5 mm	14.30	Nano Energy[22]
PEN/ITO/SnO_2/PVK/Spiro-OMeTAD/Au	19.38	500 cycles/10 mm	17.83	Adv. Funct. Mater.[23]
Cellophane/OMO/CPTA/PVK/ Spiro-OMeTAD/Au	13.00	1000 cycles/1 mm	12.45	Sol. Energy[24]
PET/ITO/SnO_2/C_{60}-SAM/PVK/ Spiro-OMeTAD/Au	17.43	1000 cycles/10 mm	13.28	ACS Energy Lett.[25]
PES/Graphene/NiO_x/PVK/PCBM/ AZO/Ag/AZO	14.00	1000 cycles/1.5%	12.60	Nano Energy[26]
MgF_2/PET/ITO/Nb_2O_5/ Spiro-OMeTAD/Au	18.40	5000 cycles/4 mm	15.20	Adv. Mater.[27]
PET/ITO/SnO_2/PVK/ Spiro-OMeTAD/Au	15.22 (30 cm^2)	1800 cycles/-	10.66	Nat. Commun.[28]
PEN/ITO/WB-SnO_2/PVK/ Spiro-OMeTAD/Ag	18.00	1000 cycles/3 mm	10.98	Adv. Funct. Mater.[29]
PEN/ITO/SnO_2/PVK/ Spiro-OMeTAD/Ag	19.51	6000 cycles/8 mm	18.53	Adv. Energy Mater.[30]
PET/ITO/NiO_x/PVK/Bis-C_{60}/Ag	14.53	100 cycles/-	11.62	ACS Nano[31]
PEN/ITO/HT-SnO_2/PVK/ PCBM/Ag	17.30	1000 cycles/14 mm	15.57	Adv. Funct. Mater.[32]
PET/ITO/NC-PEDOT:PSS/PVK PCBM/Ag	12.32 (1 cm^2)	1000 cycles/2 mm	11.46	Adv. Mater.[33]
PET/ITO/NiOx/PVK/PCBM/ BCP/Ag	15.12	5000 cycles/2.5 mm	12.85	Adv. Funct. Mater.[19]
PET/PEDOT:PSS:CFE/PVK/ PCBM/BCP/Ag	19.00	5000 cycles/3 mm	16.15	Joule[1]
**PET/ITO/PEDOT:EVA/PVK/ PCBM/Ag**	**19.87 (1 cm^2)**	**7000 cycles/3 mm**	**17.09**	**This work**

Supplementary Table 11. The sheet resistance of different conductive films.

Samples	Sheet resistance (ohm sq^{-1})
PEDOT:EVA	43.23 ± 7.43
PEDOT:PSS	-
Glass/ITO	8.45 ± 0.76
Glass/ITO/PEDOT:PSS	463.43 ± 12.23
Glass/ITO/PEDOT:EVA	12.33 ± 1.29
PET/ITO	13.82 ± 1.22
PET/ITO/PEDOT:PSS	682.12 ± 18.23
PET/ITO/PEDOT:EVA	18.47 ± 1.45

Supplementary Table 12. Mechanical parameters for finite element simulation of PEDOT:PSS-based PSCs.

**Materials**	**Thickness (μm)**	**Young's modulus (Mpa)**	**Density (ρ, g cm^{-3})**	**Poisson ratio**
ITO	0.185	840	6.80	0.25
PEDOT:PSS	0.035	471	1.39	0.32
PVK	0.35	843	4.1	0.27
$PC_{61}BM$	0.06	385	1.6	0.36
Ag	0.1	11435	10.5	0.38

The corresponding mechanical parameters and thickness of each layer are measured by the multifunctional mechanical tester and step tester.

Supplementary Table 13. Mechanical parameters for finite element simulation of PEDOT:EVA-based PSCs.

**Materials**	**Thickness (μm)**	**Young's modulus (Mpa)**	**Density (ρ, g cm^{-3})**	**Poisson ratio**
ITO	0.185	840	6.80	0.25
PEDOT:EVA	0.035	6	0.96	0.42
PVK	0.35	587	4.1	0.23
$PC_{61}BM$	0.06	385	1.6	0.36
Ag	0.1	11435	10.5	0.38

The corresponding mechanical parameters and thickness of each layer are measured by the multifunctional mechanical tester and step tester.

Supplementary Table 14. Device performance of rigid PSCs with various synthetic conditions.

Dibenzoyl peroxide (g)	3,4-ethylenedioxythiophene (g)	Benzene methyl sulfonic acid (g)	EVA (mg ml^{-1})	PCE (%)
0.85	0.60	2.50	20	20.31
1.05	0.60	2.50	20	22.16
1.25	0.60	2.50	20	20.87
1.05	0.45	2.50	20	18.32
1.05	0.75	2.50	20	19.44
1.05	0.60	1.50	20	17.75
1.05	0.60	3.50	20	20.32
1.05	0.60	2.50	5	17.32
1.05	0.60	2.50	10	19.97
1.05	0.60	2.50	30	19.65

Supplementary Table 15. Slope of the Mott-Schottky plots and doping densities of HTLs.

HTLs	slope	N_d (cm^{-3})	V_{bi} (V)	W_p (nm)
PEDOT:EVA	-5.089E14	6.409E14	0.535	12.141
PEDOT:PSS	-1.023E15	3.188E13	0.443	10.053

Supplementary Note 1.

The preparation of perovskite modules and PEDOT:EVA suspension. The preparation condition of PSMs is exactly the same as that of the small area optoelectronic devices, but the effective area is different. There are four sub-cells on a 60 × 60 mm^2 ITO/PET substrate and the length and width of one sub-cell is 60 mm and 13 mm, respectively. All the scribing techniques are performed by the rectification unit, and the dislocation of each layer is about 0.1 mm[1] (see Supplementary Figure 42).

The PEDOT:EVA suspension is prepared by miniemulsion method[2,3]. 1.05 g dibenzoyl peroxide (Sigma-Aldrich.), 0.6 g 3,4-ethylenedioxythiophene (Sigma-Aldrich.) and 2.5 g benzene methyl sulfonic acid (Sigma-Aldrich.) are mixed for 4 hours under N_2 condition. Then EVA is dissolved in chloroform at a concentration of 20 mg ml^{-1}, the solution is added dropwisely to the EDOT solution for ultrasonic in ice bath, and finally the mixed solution is continued to be ultrasonic in the ice bath for 20 hours to complete the preparation of PEDOT:EVA. The structural formula for PEDOT:EVA is shown in Supplementary Figure 43.

Next, Raman spectroscopy is used to study the interaction between PEDOT and EVA, and the results are shown in Supplementary Figure 33. In general, the $C_\alpha = C_\beta$ vibration peak at 1436 cm^{-1} is a typical characteristic peak of clew benzenoid structures of PEDOT. For the PEDOT:EVA, the characteristic peak without significant displacement and intensity changes means that the EDOT is polymerized to PEDOT. And the particle size distribution measurement (Supplementary Figure 34) proves that the size distribution and ink stability of the PEDOT:EVA solution are comparable to the commercial PEDOT:PSS. We also characterize the PEDOT:EVA and PEDOT:PSS films by X-ray photoelectron spectroscopy (XPS, Supplementary Figure 35), the appearance of binding energy intensity about 288.7 eV is the characteristic peak of carboxyl group, indicating the effective dispersion of EVA. The bandgap of PEDOT:EVA was measured by spectroelectrochemistry, which showed a 1.56 eV (λ_{onset}=795nm). And the acid-base property of PEDOT:EVA and PEDOT:PSS ink is measured by the pH-meter. The pH value of hydrophobic PEDOT:EVA is 6.49, which is more optimal than 1.20 for PEDOT:PSS, this can avoid the erosion of perovskite by acidic PEDOT:PSS (Supplementary Figure 36). Meanwhile, the device performance of rigid perovskite solar cells under different synthetic conditions is shown in the Supplementary Table 14. The PEDOT:EVA HTLs used in the manuscript is a printing ink prepared under optimal synthetic conditions.

The choice of EVA material benefits from the following three advantages:

1. For other molecules containing carboxyl group, exploring whether they interact with perovskite materials require recalculation and simulation based on the DFT molecular model, because different molecular structure, molecular conformation, functional group diversity, functional group number and steric hindrance effect will have important effects on simulation results.
2. Another feature of EVA is its remarkable adhesiveness, which is crucial for optimizing the mechanical stability of the PSCs and is also one of the starting points for selecting this material.
3. At the same time, it is also worth noting that the EVA can prepare the PEDOT ink by microemulsion method. For other materials containing carboxyl group, actual synthesis or treatment is needed to prove whether they have the potential to be applied to hole transport layers.

Supplementary Note 2.

The selection of meniscus-coating parameters. For the meniscus-coating technology, some printing parameters should be optimized, such as slot spacing (distance between the meniscus and substrate), meniscus-coating speed, Vacuum flash–assisted solution processing (VASP) treatment pressure, ratio of solution and so on. We have carries on the statistics and research to the above three basic variables, the relationship among device performance, grain size and specific parameters is shown in below. It can be clearly found that with the increase of DMSO content in the solvent (DMF), average grain size gradually is enhanced. The device PCE increases firstly and then decreases, so the DMSO content for the best efficiency is 50% (DMF:DMSO = 1:1).

As for the VASP treatment pressure, the grain size is also gradually increased with the enhancement of VASP pressure, but the device performance reaches the peak value at about 20 Pa, after that the device performance will decrease significantly. The above two conclusions can also be confirmed by XRD characterization as shown in below. The variation of peak strength and half peak width of typical perovskite crystals corresponding to 14.2° (110) and 28.4° (220) is completely consistent with the variation rule of the device performance, which confirms the correctness of the statistical diagram for meniscus-coating parameters.

The influence of meniscus-coating speed (v) on the device performance is mainly reflected in the film thickness (t). The dependence of film thickness with meniscus-coating speed follows two modes, just like the infiltration process. When the meniscus-coating speed is below 4 mm s^{-1}, the slope for the log(v) versus log(t) is about -0.97, indicating the meniscus-coating is in evaporation mode. In this mode, the ink dries immediately after the meniscus moves away from the ink surface, the solution evaporation between the meniscus and substrate determines the deposition of precursor solution. When the meniscus-coating speed is above 20 mm s^{-1}, the slope for the log(v) versus log(t) is about 0.65, which is characteristic of Landau-Levich mode theory. In this mode, the ink is still wet after meniscus-coating due to the very fast meniscus-coating speed, and additional process is required to ensure the film quality (see Supplementary Figure 44 and Supplementary Figure 45.).

Based on the above statistics, we determined the specific parameters of meniscus-coating[4]: the slot spacing is 50 μm, solvent ratio of DMF:DMSO is 1:1, VASP pressure is 20 Pa and the meniscus-coating speed is 10 mm s^{-1}.

Supplementary Note 3.

Analysis of crystal growth of perovskite based on different HTLs. In addition to the basic morphological measurements (Figure 2a and Supplementary Figure 2), we also characterize the cross-section SEM for the perovskite films based on different HTLs (Supplementary Figure 4). For the perovskite films prepared on PEDOT:PSS substrate, it is not only clearly found that the small size grains, but also many obvious transversal grain boundaries. The severe transversal grain boundaries will affect the transport of charge carriers and make the defects caused by ion diffusion (ion diffusion can be proved by the results of Tof-SIMS (Figure 4i)). Satisfactorily, perovskite films upon PEDOT:EVA have almost no transverse grain boundaries, and the perovskite grains grow oriented with larger grain size. This result can be confirmed by the XRD patterns (Supplementary Figure 3). The XRD pattern with no significant change in peak position ((110), (220)) proves that the perovskite films prepared on PEDOT:EVA did not change in lattice arrangement. While an obvious increase in peak strength indicates that better perovskite crystals are formed.

Supplementary Note 4.

LaMer diagram of crystallization kinetics. In the film forming processing of crystallizable ink, three distinct regimes of nucleation and crystal growth for perovskite precursor solution on different substrates are important and would have a crucial effect on the crystallization:

(I) pre-nucleation: with the rapid evaporation of DMF and DMSO solvent, the concentration of PbI_2 and MAI free intermediates in the precursor are promptly increased. This process continues until t_1 (Fig. 2c). In this process, the solution concentration gradually exceeds the supersaturated concentration (C_s), but remains below the minimum nucleation concentration (C_{min}^{nu}).

(II) nucleation and crystallization growth: Pb-MA-I complex in the precursor nucleates rapidly and completes the crystallization pre-growth. This process could continue from t_1 to t_2, and the solution concentration is always between the C_{min}^{nu} and the maximum nucleation concentration (C_{max}^{nu}).

(III) crystallization: the nucleation in precursor solution stops, crystallization growth until a complete dense perovskite film is formed. This process sustains from t_2 to t_3, the solution concentration is always between the C_{min}^{nu} and C_s. The crystallization growth will stop when the concentration of precursor solution is lower than C_s.

From the above description of crystallization kinetics, it can be found that the best way to increase the grain scale and quality of perovskite solar cells is to reduce the nucleation sites appropriately and extend the grain growth process. According to the classical nucleation theory, nucleation rate is controlled by a critical free energy (ΔG_c), which represents the free energy required for nuclei to be stable without being dissolved in the solution. And the critical free energy is also defined as activation energy of nucleation, which is used to describe nucleation rate by the Arrhenius type equation[5-8]:

$$\frac{dN}{dt} = A\,exp\left(-\frac{\Delta G_C}{k_B T}\right) \quad (1)$$

Where t is time, N is number of nuclei, so dN/dt is the nucleation rate, A is pre-exponential factor, k_b is the Boltzmann's constant, and T is temperature. In addition to affecting the crystallization rate, the crystal free energy (ΔG_c) itself is also affected by variety of factors and can be defined as:

$$\Delta G_C = \frac{16\pi\gamma^3 v^2}{3K_B^2 T^2 (ln\,S)^2} \quad (2)$$

Where the γ is the surface energy, v is the molar volume, S is supersaturation of solution. It can be seen from the equation (1) and (2) that ΔG_c is affected by the above four parameters, and then has a significant impact on the nucleation rate of crystallization. In general, larger surface energy means the greater gibbs free energy (ΔG_c), lower crystallization rate and reduces nucleation site, which can be derived from the equation (1) and (2) for this transformation relation. Meanwhile, shorter nucleation time also provides more time for grain growth to ensure grain quality and form a perfect perovskite film (This phenomenon corresponds to the results of SEM images and 2D-XRD measurements in Fig. 2).

Supplementary Note 5.

Calculation results using the AIMD-RDF code. Density function theory calculation is performed by using the CP2K package.[9] PBE functional[10] with Grimme D3 correction[11] is used to describe the system. Kohn-Sham DFT has been used as the electronic structure method in the framework of the Gaussian and plane waves method.[12,13] The Goedecker-Teter-Hutter (GTH) pseudopotentials[14,15], DZVP-MOLOPT-GTH basis sets[12] are utilized to describe the molecules. A plane-wave energy cut-off of 500 Ry has been employed. We perform ab-initio molecular dynamics (AIMD) for the perovskite surface.

We have considered two terminations of the surface, PbI_2-termination and MAI-termination, as shown in the Fig. 2g and Supplementary Figure 6-9. We place four EVA molecules and two PEDOT:PSS molecules on the top of two surface terminations to study the interaction between perovskite and molecules. The NVT ensemble has been performed at 300K using Canonical sampling through velocity rescaling[16] with the time step of 0.5 fs. The simulation is carried out in a three-dimensional periodic boundary box of 17.390 × 17.390 × 50 $Å^3$ for $MAPbI_3$ surface. We equilibrate the system for about 5 ps and a time length of 15 ps used to analyze and calculate the radial distribution function (RDF) of the molecules when interacting with perovskite surface.

Supplementary Note 6.

The intrinsic properties of PEDOT:EVA films. The optical transmittance of PET/ITO/PEDOT:EVA or PEDOT:PSS (the thickness of PET/ITO transparent electrode is 0.125 mm) and glass/ITO/PEDOT:EVA or PEDOT:PSS is shown in Supplementary Figure 38. Benefiting by the ultra-thin transparent electrode materials, the difference in the final transmittance for different substrate materials is not obvious, which means that the substrate materials have a little effect on the light absorption of perovskite films. The conductivity of PEDOT:EVA and ITO/PEDOT:EVA is then characterized, and the results are shown in Supplementary Table 9. It can be found that the conductivity of PEDOT:EVA film is better than PEDOT:PSS, and due to the excellent conductivity of ITO electrode, the conductivity of ITO/PEDOT:EVA is not significantly different from ITO/PEDOT:PSS, proving that the influence of different substrate materials on the performance of the PSCs is limited. Finally, the PEDOT:EVA and PEDOT:PSS films are morphologic characterized by AFM, as shown in Supplementary Figure 37. Different from the typical morphology of PEDOT:PSS, the PEDOT:EVA films appear an obvious fibrous structure, which is related to the significant reduction of PSS content on the film surface. In general, the overall performance of PEDOT:EVA film is better than PEDOT:PSS, which is conducive to the improvement of device performance. Meanwhile, more importantly, the optimization of perovskite crystal quality caused by PEDOT:EVA films is also not negligible.

Supplementary Note 7.

Mott-Schottky plots for PSCs. The intersection of X-axis and the straight-line in the cures is defined as the flat-band potential (V_{bi}). Excluding the constant parameters during the device preparation, only the HTLs of the PSCs has changed in this work, so the V_{bi} of devices is directly correlated to different HTLs.

We also calculate the doping density by the Mott-Schottky equation:

$$\frac{1}{C^2} = \frac{2}{\varepsilon\varepsilon_0 q A^2 N}(V_{bi} - V) \tag{3}$$

and

$$W_p = \left[\frac{2\varepsilon\varepsilon_0}{qN}(V - V_{bi})\right]^{0.5} \tag{4}$$

where the C is capacitance, $\mathcal{E}$ is the dielectric constant (46.9), $\mathcal{E}_0$ is the permittivity of free space, q is the elementary charge, A is the effective area (1.01 cm^2), N is the doping density of the semiconductor, V is the applied bias and W_p is depletion zone width. The calculation results are shown in Supplementary Table 15. For the PSCs devices, the improved doping density (N_d) will optimize the charge collection efficiency obviously and the depletion zone reflects a bulk resistance from perovskite to HTL, which can be expressed as depletion zone width (W_p)[17,18].

Supplementary Note 8.

Analysis of HTLs mobility and charge transmission performance. Electrical impedance spectroscopy is used to detect contact resistance information at the interface between perovskite and HTLs. The series resistance (R_s) and recombination resistance (R_{rec}) in devices with the structure of ITO/HTLs/perovskite/PCBM/BCP/Au are evaluated. The Nyquist plots for all devices are shown in Supplementary Figure 17. The R_s value for the devices prepared on PTDOT: EVA is 5.63 Ω cm^{-2}, which is smaller than 6.98 Ω cm^{-2} for the PEDOT:PSS devices, along with the corresponding R_{rec} values changing from 3150 to 2314 Ω cm^{-2}. The lower R_s value and higher R_{rec} value indicate that the series resistance at the interface between perovskite and HTLs will be significantly suppressed by the EVA modification.

Next, the mobility of PEDOT: EVA and PEDOT:PSS films (the device structure prepared by meniscus-coating is glass/ITO/HTLs/perovskite/Au) is measured by the space-charge-limited-current module (SCLC), the results are shown in Supplementary Figure 18 and Supplementary Table 5. The mobility of perovskite films upon PEDOT:EVA is calculated to be 1.19×10^{-4} cm^2 V^{-1} s^{-1}, which is much higher than that of reference (9.35×10^{-5} cm^2 V^{-1} s^{-1}). The higher mobility value can better match with the electron mobility of PCBM, and further realize the charge carrier migration balance devices[19]. The above characterization results indicate that PEDOT:EVA HTLs can significantly improve the optoelectronic properties of the perovskite films, which is due to the enhanced charge mobility, reduced trap state density and the optimized contact resistance at the charge extraction layer. These improvements are beneficial for more perfect V_{oc} and FF in the PSCs[20].

Supplementary Note 9.

The mechanical stability of ITO flexible transparent electrode. The flexible transparent electrode materials for the preparation of flexible devices are ITO/PET substrate with a thickness of 0.125 mm. It can be found from the SEM images that ITO substrates covered with PEDOT:EVA verify the excellent mechanical stability under various bending radius compared with those covered with PEDOT:PSS, and no obvious micron-scale cracks appear on the surface of the film, which is due to the bonding property of PEDOT:EVA material (Supplementary Figure 24 and Supplementary Figure 25). Meanwhile, in order to prove the substantive characteristics of PEDOT:EVA cohesiveness, we conducted the bond performance measurement. The PET film bonded with PEDOT:EVA material showed distinguished tensile performance, which is similar to the result in Figure1b, and this is significantly better than PEDOT:PSS ink (Supplementary Figure 23). To determine the mechanical performance, we also summary the recent representative reports of flexible PSCs with different approaches[1,19,21-33], and the results are shown in Supplementary Figure 31 and Supplementary Table 10.

To illustrate this phenomenon, We construct a mechanical model with structure of ITO/HTL (PEDOT:EVA or PEDOT:PSS)/perovskite/PCBM/Ag. By combining the Young's Modulus, poisson's ratio and film thickness for different layers, the stress distribution of the whole devices in the bending process is deeply analyzed and simulated (As shown in Supplementary Figure 39 and Supplementary Table 12 and Table 13). After applying the stress, we simulate the overall mechanical distribution for the corresponding ITO and perovskite layers respectively, and the results are shown in Supplementary Figure 40. It can be clearly found that PEDOT:EVA buffer layer can significantly reduce the overall stress distribution for the films in bending process, no matter for perovskite and ITO layers. This is due to the lower Young's Modulus (The mechanical parameters and thickness of each layer were measured by the multifunctional mechanical tester and step tester respectively) and density for the PEDOT:EVA film and optimized poisson ratio, compared with PEDOT:PSS film, which provide a preliminary explanation for the improved mechanical stability of the ITO film.

In addition, we explain the contribution of PEDOT:EVA layer to the mechanical stability of ITO layer from the mechanical structure analysis. As a conductive film, ITO conducts electricity by the carrier migration between the crystal lattice. When external forces are applied to the ITO film and obvious deformation occurs, once the inherent microstructure of the film is destroyed, the carrier migration channel will be affected, which is manifested as a sharp deterioration in electrical conductivity macroscopically. When the strain variable increases to a certain value, the structure of the ITO film will be seriously damaged, and its resistance value will have an obvious mutation, which is called critical strain[34-36]. Previous reports have shown that the binding ability between the buffer layer and ITO layer will significantly affect the bending resistance for the brittle ITO layer[37,38]. Due to the adhesiveness, PEDOT:EVA shows impressive binding ability with perovskite and ITO layers through the adsorption and Van Der Waal force of PEDOT:EVA glue, which is also crucial for improving the mechanical stability of ITO layer. In order to explore the change of critical strain, we conducted in-situ measurements for the original ITO/PEDOT:EVA and ITO/PEDOT:PSS films (R_0) with the ITO/PEDOT:EVA and ITO/PEDOT:PSS films at different

bending angles (R). The results are shown in Supplementary Figure 41. For the ITO film coated with PEDOT:EVA, after 1500 bending cycles, the conductive property does not change significantly at the bending angle from 10 to 70 degree, which means the structure of ITO film is not damaged. However, for the ITO films coated with PEDOT:PSS, the electrical conductivity not only degrades obviously, but also increases with the increase of bending angle, suggesting the film structure is destroyed.

Meanwhile, in order to further explain the optimization of mechanical stability, we also calculate the mechanical mismatch coefficient. The damage condition of film mainly depends on the material property, but once the damage occurs, its further extension will depend on the release rate of strain energy in the film[39-41]. Therefore, we analyze the above experiment results by the Dundurs coefficient (α and β).

$$\alpha = \frac{\bar{E} - \overline{E_s}}{\bar{E} + \overline{E_s}} \quad (5)$$

$$\beta = \frac{\bar{E}\left(\frac{1-2v_s}{1-v_s}\right) - \overline{E_s}\left(\frac{1-2v}{1-v}\right)}{2(\bar{E} + \overline{E_s})} \quad (6)$$

Where E and E_s are the Young's Modulus of the PEDOT:EVA (PEDOT:PSS) and ITO layers, v and v_s are the poisson's ratio of the PEDOT:EVA (PEDOT:PSS) and ITO layers. In addition, $\overline{E}$ and $\overline{E_s}$ can be calculated by the following formula: $\overline{E} = E/(1-v^2)$ and $\overline{E_s} = E_s/(1-v_s^2)$. In the absence of energy loss, the value of β tends to zero and the α describes the mismatch degree of the Young's Modulus between the HTL and ITO layers, with values ranging from -1 to 1. The lower α value implies a worse mismatch degree and indicates a more effective inhibition for the microcracks. Due to the more optimized Young's Modulus of PEDOT:EVA, the ITO/PEDOT:EVA film (-0.703) exhibits superior mechanical stability, compared with the ITO/PEDOT:PSS film (-0.506). We also calculated the strain energy stored in per unit width for the films before fracture (G_0) and the strain energy release rate (G_{ten}). The calculation of G_0 and G_{ten} are shown below.

$$G_0 = \frac{1}{2}\bar{E}h\varepsilon^2 \quad (7)$$

$$G_{ten} = G_0 g(\alpha, \beta) \quad (8)$$

Where h is the film thickness, ε is the strain at a certain bending radius, $g(\alpha, \beta)$ is a function which is proportional to α. For the different films with similar thickness and bending radius, lower Young's Modulus means lower G_0. In combination with the direct proportional relationship between $g(\alpha, \beta)$ and α, these will lead to a lower G_{ten}. Excessive strain energy release rate will not limit the extension of the damaged area in the film, on the contrary, the damaged area will be difficult to extend on the interface. This is consistent with the change of conductivity and surface morphology for the ITO/PEDOT:EVA and ITO/PEDOT:PSS films under 4500 bending cycles.

Supplementary Note 10.

Analysis of the film's resistance to strain and specific parameters. The ratio of stress and strain is elastic modulus. Young's modulus is a physical quantity that describes the material's resistance to deformation, which is one of the most common elastic modulus. The value of Young's modulus reflects the difficulty of elastic deformation for the materials. Rigidity modulus is the ratio of shear stress to strain, the larger modulus means that the material is more rigid and vice versa. Rigidity modulus can be calculated by the following formula:

$$G = E/(2\cdot(1+v)) \qquad (9)$$

where G is the rigidity modulus, E is the Young's modulus and v is the poisson ratio. The Young's modulus can be measured by AFM mechanical model, and the values are 139 MPa and 258 MPa for the PET/ITO/PEDOT:EVA and PET/ITO/PEDOT:PSS substrates, respectively. The poisson ratios based on the above conditions are 0.36 and 0.25. By this way, the rigidity modulus of different HTLs can be easily calculated, with specific values of 51.10 MPa and 67.21 MPa. This result indicates that PEDOT:EVA is more flexible than PEDOT:PSS.

Supplementary Note 11.

The mechanism of "vertebrae" bionics. The mechanism of "vertebrae" bionics mainly comes from two aspects (structure bionics and crystal bionics). In terms of structure bionics, articular cartilage is between the two vertebrae, either hyaline cartilage or fibrous cartilage, which is similar to the characteristics of fibrous PEDOT:EVA layer (Supplementary Figure 37). Social activities in a person's life are inseparable from the normal function of articular cartilage. Articular cartilage can ensure that the vertebrae are not damaged, one of the reasons is that the articular cartilage has the function of the force absorption. Articular cartilage can distribute the force evenly and enlarge the bearing surface. This not only maximizes the mechanical load, but also protects the vertebrae from damage, which is due to the elasticity and adhesiveness of the articular cartilage. This feature is also similar to the PEDOT:EVA attribute (Supplementary Figure 23). Therefore, we compare the structure of ITO/PEDOT:EVA/PVK to the flexural structure of vertebrae/cartilage/vertebrae, and considered it reasonable.

In terms of crystal bionics, we regard the nucleation process of perovskite precursor and the growth process of vertebrae as the bionic key point. Vertebrae growth is a mineralization process that involves four main steps:

1. Preorganization of organic matter: the insoluble organic matter in organisms forms an organized microreactive environment before mineral deposition, which determines the location of inorganic matter nucleation and the function of mineral formation.
2. Interface molecular recognition: under the control of the assembled organic macromolecules, inorganic materials nucleate at the organic-inorganic interface in solution by electrostatic force, chelation, hydrogen bond, van der Waals force, etc.
3. Growth modulation: the morphology, size, orientation and structure of the crystals are regulated by the organic matter of organisms during the growth of inorganic mineral phases, and subunits are initially assembled.
4. Crystal epitaxial growth.

It can be clearly found from the above description, for the growth of vertebrae, the hydrophobic organic interface, organic-inorganic interaction sites and crystal growth conditions are critical. This nucleation regulation process is very similar to the regulation mechanism of PEDOT:EVA HTL on nucleation in the manuscript. Therefore, we call the regulation of PEDOT:EVA on the perovskite crystallization as crystal bionics.

Supplementary References

1. Hu, X. *et al.* A Mechanically Robust Conducting Polymer Network Electrode for Efficient Flexible Perovskite Solar Cells. *Joule* **3**, 2205-2218 (2019).
2. Xie, C. *et al.* Overcoming Microstructural Limitations in Water Processed Organic Solar Cells by Engineering Customized Nanoparticulate Inks. *Adv. Energy Mater.* **8**, 1702857 (2018).
3. Xie, C. *et al.* Overcoming efficiency and stability limits in water-processing nanoparticular organic photovoltaics by minimizing microstructure defects. *Nat. Commun.* **9**, 5335 (2018).
4. Deng, Y. *et al.* Surfactant-controlled ink drying enables high-speed deposition of perovskite films for efficient photovoltaic modules. *Nat. Energy* **3**, 560 (2018).
5. Chiang, C. H. & Wu, C. G. A Method for the Preparation of Highly Oriented MAPbI3 Crystallites for High Efficiency Perovskite Solar Cells to Achieve an 86% Fill Factor. *ACS Nano* **12**, 10355-10364 (2018).
6. Sanchez, S., Hua, X., Phung, N., Steiner, U. & Abate, A. Flash Infrared Annealing for Antisolvent-Free Highly Efficient Perovskite Solar Cells. *Adv. Energy Mater.* **8**, 1702915 (2018).
7. Hu, H. *et al.* Room-Temperature Meniscus Coating of >20% Perovskite Solar Cells: A Film Formation Mechanism Investigation. *Adv. Funct. Mater.* **29**, 1900092 (2019).
8. Huang, F., Li, M., Siffalovic, P., Cao, G. & Tian, J. From scalable solution fabrication of perovskite films towards commercialization of solar cells. *Energy Environ. Sci.* **12**, 518-549 (2019).
9. Hutter, J., Iannuzzi, M., Schiffmann, F. & Vondele, J. V. Cp2k: atomistic simulations of condensed matter systems. *WIREs Comput. Mol. Sci.* **4**, 15–25 (2014).
10. Perdew, J. P., Burke, K. & Ernzerhof, M. Generalized gradient approximation made simple. *Phys. Rev. Lett.* **77**, 3865 (1996).
11. Grimme, S. Semiempirical gga-type density functional constructed with a long-range dispersion correction. *J. Comput. Chem.* **27**, 1787–1799 (2006).
12. Vondele, J. V. & Hutter J. Gaussian basis sets for accurate calculations on molecular systems in gas and condensed phases. *J. Chem. Phys.* **127**, 114105 (2007).
13. Vondele, J. V. *et al.* Quickstep: Fast and accurate density functional calculations using a mixed gaussian and plane waves approach. *Comput. Phys. Commun.* **167**, 103–128 (2005).
14. Goedecker, S., Teter, M. & Hutter, J. Separable dual-space gaussian pseudopotentials. *Phys. Rev. B*, **54**, 1703 (1996).
15. Hartwigsen, C., Gœdecker, S. & Hutter, J. Relativistic separable dual-space Gaussian pseudopotentials from h to rn. *Phys. Rev. B*, **58**, 3641 (1998).
16. Bussi, G., Donadio, D. & Parrinello, M. Canonical sampling through velocity rescaling. *J. Chem. Phys.* **126,** 014101 (2007).
17. Xie, L. *et al.* Low-Cost Coenzyme Q10 as Efficient Electron Transport Layer for Inverted Perovskite Solar Cells. *J. Mater. Chem. A* **7**, 18626-18633 (2019).
18. Wang, Y., Zhang, T., Kan, M. & Zhao, Y. Bifunctional Stabilization of All-Inorganic α-CsPbI3 Perovskite for 17% Efficiency Photovoltaics. *J. Am. Chem. Soc.* **140**, 12345-12348 (2018).
19. Huang, Z. *et al.* Water-Resistant and Flexible Perovskite Solar Cells via a Glued Interfacial Layer. *Adv. Funct. Mater.* **29**, 1902629 (2019).
20. Liu, C. *et al.* Enhanced Hole Transportation for Inverted Tin-Based Perovskite Solar Cells with High

Performance and Stability. *Adv. Funct. Mater.* **29**, 1808059 (2019).

21. Yang, D. *et al.* High efficiency planar-type perovskite solar cells with negligible hysteresis using EDTA-complexed SnO_2. *Nat. Commun.* **9**, 3239 (2018).
22. Wang, C. *et al.* Compositional and morphological engineering of mixed cation perovskite films for highly efficient planar and flexible solar cells with reduced hysteresis. *Nano Energy* **35**, 223-232 (2017).
23. Wu, C. *et al.* $FAPbI_3$ Flexible Solar Cells with a Record Efficiency of 19.38% Fabricated in Air via Ligand and Additive Synergetic Process. *Adv. Funct. Mater.* **29**, 1902974 (2019).
24. Li, H. *et al.* Ultraflexible and biodegradable perovskite solar cells utilizing ultrathin cellophane paper substrates and TiO2/Ag/TiO2 transparent electrodes. *Sol. Energy* **188**, 158-163 (2019).
25. Wang, C. *et al.* Water Vapor Treatment of Low-Temperature Deposited SnO_2 Electron Selective Layers for Efficient Flexible Perovskite Solar Cells. *ACS Energy Lett.* **2**, 2118-2124 (2017).
26. Tran, V. D. *et al.* Transfer-free graphene electrodes for super-flexible and semi-transparent perovskite solar cells fabricated under ambient air. *Nano Energy* **65**, 104018 (2019).
27. Feng, J. *et al.* Record Efficiency Stable Flexible Perovskite Solar Cell Using Effective Additive Assistant Strategy. *Adv. Mater.* **30**, 1801418 (2018).
28. Bu, T. *et al.* Universal passivation strategy to slot-die printed SnO_2 for hysteresis-free efficient flexible perovskite solar module. *Nat. Commun.* **9**, 4609 (2018).
29. Chen, C. *et al.* Solvent-Assisted Low-Temperature Crystallization of SnO_2 Electron-Transfer Layer for High-Efficiency Planar Perovskite Solar Cells. *Adv. Funct. Mater.* **29**, 1900557 (2019).
30. Huang, K. *et al.* High-Performance Flexible Perovskite Solar Cells via Precise Control of Electron Transport Layer. *Adv. Energy Mater.* **9**, 1901419 (2019).
31. Zhang, H. *et al.* Pinhole-Free and Surface-Nanostructured NiO_x Film by Room-Temperature Solution Process for High-Performance Flexible Perovskite Solar Cells with Good Stability and Reproducibility. *ACS Nano* **10**, 1503-1511 (2016).
32. Liu, C. *et al.* Hydrothermally Treated SnO_2 as the Electron Transport Layer in High-Efficiency Flexible Perovskite Solar Cells with a Certificated Efficiency of 17.3%. *Adv. Funct. Mater.* **29**, 1807604 (2019).
33. Hu, X. *et al.* Wearable Large-Scale Perovskite Solar-Power Source via Nanocellular Scaffold. *Adv. Mater.* **29**, 1703236 (2017).
34. Li, T. C. & Lin, J. F. Fatigue life study of ITO/PET specimens in cyclic bending tests. *J. Mater. Sci-Mater. El.* **26**, 250-261 (2015).
35. Alzoubi, K., Hamasha, M. M., Lu, S. & Sammakia, B. Bending Fatigue Study of Sputtered ITO on Flexible Substrate. *J. Disp. Technol.* **7**, 593-600 (2011).
36. Li, T. C., Han, C. F., Chen, K. T. & Lin, J. F. Fatigue Life Study of ITO/PET Specimens in Terms of Electrical Resistance and Stress/Strain Via Cyclic Bending Tests. *J. Disp. Technol.* **9**, 577-585 (2013).
37. Lan, Y. F., Peng, W. C., Lo, Y. H. & He, J. L. Durability under mechanical bending of the indium tin oxide films deposited on polymer substrate by thermionically enhanced sputtering. *Org. Electron.* **11**, 670-676 (2010).
38. Lin, H. K., Chiu, S. M., Cho, T. P. & Huang, J. C. Improved bending fatigue behavior of flexible PET/ITO film with thin metallic glass interlayer. *Mater. Lett.* **113**, 182-185 (2013).
39. Hutchinson, J. W. & Suo, Z. Mixed mode cracking in layered materials. *Adv. Appl. Mech.* **29**, 63-191 (1992).

40. Chen, Z. & Cotterell, B. A mechanical assessment of flexible optoelectronic devices. *Thin Solid Films* **394**, 201-205 (2001).
41. Leterrier, Y. *et al.* Mechanical integrity of transparent conductive oxide films for flexible polymer-based displays. *Thin Solid Films* **460**, 156-166 (2004).
