# Supplementary material for: Bio-inspired vertebral design for scalable and flexible perovskite solar cells
Source: Nat Commun. 2020 Jun 15;11:3016. doi: 10.1038/s41467-020-16831-3 (PMC7295992; doi:10.1038/s41467-020-16831-3)
Supplement: Supplementary file 3 — Reporting Summary [file 41467_2020_16831_MOESM3_ESM.pdf]

nature research

Corresponding author(s): Yiwang Chen, Yanlin Song, Xiaotian Hu

Solar Cells Reporting Summary

Nature Research wishes to improve the reproducibility of the work that we publish. This form is intended for publication with all accepted papers reporting the characterization of photovoltaic devices and provides structure for consistency and transparency in reporting. Some list items might not apply to an individual manuscript, but all fields must be completed for clarity.

For further information on Nature Research policies, including our data availability policy, see Authors & Referees.

ü Experimental design

Please check: are the following details reported in the manuscript?

1. Dimensions

Item	Yes	No	Details
Area of the tested solar cells	x		The small area of solar cells is 2.25 cm2, and large area device is 36 cm2 (Methods)
Method used to determine the device area	x		The active area is 1.01 cm2 and 31.2 cm2, determined by the aperture shade mask (Methods)

2. Current-voltage characterization

Item	Yes	No	Details
Current density-voltage (J-V) plots in both forward and backward direction	x		Fig. 3e and Supplementary Fig. 14
Voltage scan conditions<br>*For instance: scan direction, speed, dwell times*	x		J-V curves were measured with a scanning rate of 100 mV/s (voltage step of 20 mVand delay time of 200 ms) (Methods, device haracterization)
Test environment<br>*For instance: characterization temperature, in air or in glove box*	x		Performance measurements were carried in nitrogen environment (Methods)
Protocol for preconditioning of the device before its characterization	x		No preconditioning was used.
Stability of the J-V characteristic<br>*Verified with time evolution of the maximum power point or with the photocurrent at maximum power point; see ref. 7 for details.*	x		Maximum power point tracking (Supplementary Fig.11 and Supplementary Fig.20)

3. Hysteresis or any other unusual behaviour

Item	Yes	No	Details
Description of the unusual behaviour observed during the characterization	x		Optimized cells showed negligible hysteresis.
Related experimental data	x		Fig. 3d and Supplementary Fig. 12

4. Efficiency

Item	Yes	No	Details
External quantum efficiency (EQE) or incident photons to current efficiency (IPCE)	x		Fig. 3e and Supplementary Fig. 14
A comparison between the integrated response under the standard reference spectrum and the response measure under the simulator	x		Fig. 3e and Supplementary Fig. 14
For tandem solar cells, the bias illumination and bias voltage used for each subcell		x	*Explain why this information is not reported/not relevant.*

5. Calibration

Item	Yes	No	Details
Light source and reference cell or sensor used for the characterization	x		Enli Tech simulator is used for the measurements (Methods).
Confirmation that the reference cell was calibrated and certified	x		The light intensity was calibrated by reference solar cell by Enli Tech.

Calculation of spectral mismatch between the reference cell and the devices under test

- [x] Yes
- [ ] No

Mismatch factor of 1 was used in our measurements.

6. Mask/aperture

Size of the mask/aperture used during testing

- [x] Yes
- [ ] No

1.01 cm2 and 31.2 cm2 (Methods, device characterization).

Variation of the measured short-circuit current density with the mask/aperture area

- [x] Yes
- [ ] No

We always tested device performance at the same aperture area.

7. Performance certification

Identity of the independent certification laboratory that confirmed the photovoltaic performance

- [x] Yes
- [ ] No

The device efficiency certification report for perovskite solar cell module with a 36 cm2 effective area (by National Institute of Metrology, China).

A copy of any certificate(s)
Provide in Supplementary Information

- [x] Yes
- [ ] No

Supplementary Fig. 21

8. Statistics

Number of solar cells tested

- [x] Yes
- [ ] No

At least 50 devices under each strain condition were tested (Supplementary Tab.6 and Supplementary Tab. 7).

Statistical analysis of the device performance

- [x] Yes
- [ ] No

Supplementary Tab.6 and Supplementary Tab. 7

9. Long-term stability analysis

Type of analysis, bias conditions and environmental conditions
For instance: illumination type, temperature, atmosphere humidity, encapsulation method, preconditioning temperature

- [x] Yes
- [ ] No

Depicted in Supplementary Fig. 32
