# Supplementary material for: Bio-inspired vertebral design for scalable and flexible perovskite solar cells
Source: Nat Commun. 2020 Jun 15;11:3016. doi: 10.1038/s41467-020-16831-3 (PMC7295992; doi:10.1038/s41467-020-16831-3)
Supplement: Supplementary file 4 — Description of Additional Supplementary Files [file 41467_2020_16831_MOESM4_ESM.pdf]

Description of Additional Supplementary Files

Supplementary Movie 1. The video shows the perovskite solar power source can charge multi-function smart-watch during arm movement (~20 klx solar irradiance).

Supplementary Movie 2. The video shows the perovskite solar power source can meet the requirements of normal operation for the electric fan (~20 klx solar irradiance).

Supplementary Movie 3. The dynamic simulation of the interaction between PbI_2 and EVA, proving that there is indeed a strong interaction between PbI_2 and EVA.

Supplementary Movie 4. The dynamic simulation of the interaction between MAI and EVA, proving that there is no interaction between MAI and EVA.

Supplementary Movie 5. The dynamic simulation of the interaction between PbI_2 and PSS, proving that there is indeed a weak interaction between PbI_2 and PSS.

Supplementary Movie 6. The dynamic simulation of the interaction between MAI and PSS, proving that there is no interaction between MAI and PSS.
